# Problem-solving training as an active ingredient of treatment for youth depression: a scoping review and exploratory meta-analysis

**DOI:** 10.1186/s12888-021-03260-9

**Published:** 2021-08-24

**Authors:** Karolin R. Krause, Darren B. Courtney, Benjamin W. C. Chan, Sarah Bonato, Madison Aitken, Jacqueline Relihan, Matthew Prebeg, Karleigh Darnay, Lisa D. Hawke, Priya Watson, Peter Szatmari

**Affiliations:** 1 Cundill Centre for Child and Youth Depression, Centre for Addiction and Mental Health (CAMH), 80 Workman Way, Toronto, ON M6J 1H4 Canada; 2 Evidence Based Practice Unit, University College London and Anna Freud National Centre for Children and Families, London, UK; 3 Department of Psychiatry, University of Toronto, Toronto, ON Canada; 4 Independent Family Doctor, Toronto, ON Canada; 5 Hospital for Sick Children, Toronto, ON Canada

**Keywords:** Problem solving, Depression, Adolescence, Youth, Active ingredient

## Abstract

**Background:**

Problem-solving training is a common ingredient of evidence-based therapies for youth depression and has shown effectiveness as a versatile stand-alone intervention in adults. This scoping review provided a first overview of the evidence supporting problem solving as a mechanism for treating depression in youth aged 14 to 24 years.

**Methods:**

Five bibliographic databases (APA PsycINFO, CINAHL, Embase, MEDLINE, Web of Science) and the grey literature were systematically searched for controlled trials of stand-alone problem-solving therapy; secondary analyses of trial data exploring problem-solving-related concepts as predictors, moderators, or mediators of treatment response within broader therapies; and clinical practice guidelines for youth depression. Following the scoping review, an exploratory meta-analysis examined the overall effectiveness of stand-alone problem-solving therapy.

**Results:**

Inclusion criteria were met by four randomized trials of problem-solving therapy (524 participants); four secondary analyses of problem-solving-related concepts as predictors, moderators, or mediators; and 23 practice guidelines. The only clinical trial rated as having a low risk of bias found problem-solving training helped youth solve personal problems but was not significantly more effective than the control at reducing emotional symptoms. An exploratory meta-analysis showed a small and non-significant effect on self-reported depression or emotional symptoms (Hedges’ g = − 0.34; 95% CI: − 0.92 to 0.23) with high heterogeneity. Removing one study at high risk of bias led to a decrease in effect size and heterogeneity (g = − 0.08; 95% CI: − 0.26 to 0.10). A GRADE appraisal suggested a low overall quality of the evidence. Tentative evidence from secondary analyses suggested problem-solving training might enhance outcomes in cognitive-behavioural therapy and family therapy, but dedicated dismantling studies are needed to corroborate these findings. Clinical practice guidelines did not recommend problem-solving training as a stand-alone treatment for youth depression, but five mentioned it as a treatment ingredient.

**Conclusions:**

On its own, problem-solving training may be beneficial for helping youth solve personal challenges, but it may not measurably reduce depressive symptoms. Youth experiencing elevated depressive symptoms may require more comprehensive psychotherapeutic support alongside problem-solving training. High-quality studies are needed to examine the effectiveness of problem-solving training as a stand-alone approach and as a treatment ingredient.

**Supplementary Information:**

The online version contains supplementary material available at 10.1186/s12888-021-03260-9.

## Background

Depressive disorders are a common mental health concern in adolescence [1–3] and associated with functional impairment [4] and an increased risk of adverse mental health, physical health, and socio-economic outcomes in adulthood [5–8]. Early and effective intervention is needed to reduce the burden arising from early-onset depression. Several psychotherapies have proven modestly effective at reducing youth depression, including cognitive-behavioural therapy (CBT) and interpersonal therapy (IPT) [9, 10]. Room for improvement remains; around half of youth do not show measurable symptom reduction after an average of 30 weeks of routine clinical care for depression or anxiety [11]. One barrier to greater impact is a lack of understanding of which treatment ingredients are most critical [12, 13]. Identifying the “active ingredients” that underpin effective approaches, and understanding when and for whom they are most effective is an important avenue for enhancing impact [13]. Distilling interventions to their most effective ingredients while removing redundant content may also help reduce treatment length and cost, freeing up resources to expand service provision. Given that youth frequently drop out of treatment early [14], introducing the most effective ingredients at the start may also help improve outcomes.

**Fig. 1:**
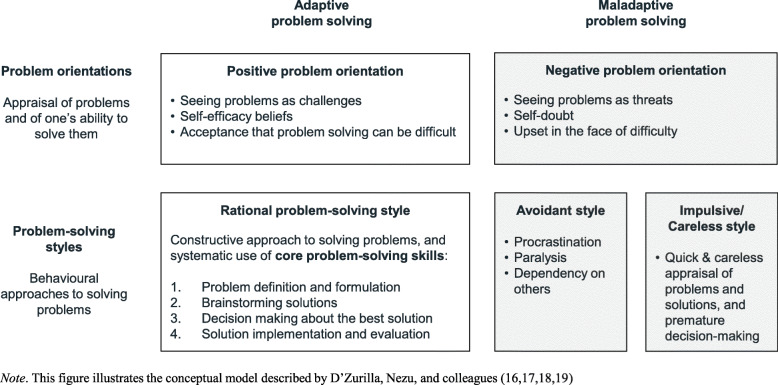
Dimensions of Problem-Solving (PS) Ability

One common ingredient in the treatment of youth depression is problem-solving (PS) training [15]. Problem solving in real-life contexts (also called *social* problem solving) describes “the self-directed process by which individuals attempt to identify [ …] adaptive coping solutions for problems, both acute and chronic, that they encounter in everyday living” (p.8) [16]. Within a relational/problem-solving model of stress and well-being, mental health difficulties are viewed as the result of maladaptive coping behaviours that cannot adequately safeguard an individual’s well-being against chronic or acute stressors [17]. According to a conceptual model developed by D’Zurilla and colleagues ([16, 17, 18, 19]; see Fig. 1), effective PS requires a constructive and confident attitude towards problems (i.e., a positive *problem orientation*), and the ability to approach problems rationally and systematically (i.e., *rational PS style*). Defeatist or catastrophizing attitudes (i.e., a *negative problem orientation*), passively waiting for problems to resolve (i.e., *avoidant style*), or acting impulsively without thinking through possible consequences and alternative solutions (i.e., *impulsive/careless style*) are considered maladaptive [16, 18, 20]. Empirical studies suggest maladaptive PS is associated with depressive symptoms in adolescents and young adults [21–25].

Problem-Solving Therapy (PST) is a therapeutic approach developed by D’Zurilla and Goldfried [26] in the 1970s, to alleviate mental health difficulties by improving PS ability. Conceptually rooted in Social Learning Theory [27], PST aims to promote adaptive PS by helping clients foster an optimistic and self-confident attitude towards problems (i.e., a positive problem orientation), and by helping them develop and internalize four core PS skills: (a) defining the problem; (b) brainstorming possible solutions; (c) appraising solutions and selecting the most promising one; (d) implementing the preferred solution and reflecting on the outcome ([16–19]; see Fig. 1). PST is distinct from Solution-Focused Brief Therapy (SFBT), which has different conceptual roots and emphasizes the construction of solutions over the in-depth formulation of problems [28].

PS training is also a common ingredient of other psychosocial depression treatments [15, 20], such as CBT and Dialectical Behaviour Therapy (DBT) [15, 29–32] that typically focus on strengthening PS skills rather than problem orientation [20]. In IPT, PS training focuses on helping youth understand and resolve relationship problems [29, 30, 33, 34]. PS training is also a common component of family therapy [35], cognitive reminiscence therapy [36], and adventure therapy [37]. The extent to which PS training in these contexts follows the conceptual model by D’Zurilla and colleagues varies. Hereafter, we will use the term PST (“Problem-Solving Therapy”) where problem-solving training constitutes a stand-alone intervention; and we will use the term “PS training” where it is mentioned as a part of other therapies or discussed more broadly as an active ingredient of treatment for youth depression.

Meta-analyses considering over 30 randomized control trials (RCTs) of stand-alone PST for adult depression suggest it is as effective as CBT and IPT, and more effective than waitlist or attention controls [38–40]. PST has been applied with children, adolescents, and young adults [41–46], but dedicated manuals for different developmental stages are not readily available. In an assessment of fit between evidence-based therapy components and everyday coping skills used by school children, PS skills were the third most frequently endorsed skill set in terms of frequency of habitual use and perceived effectiveness, suggesting these skills are highly transferable and relevant to youth [47]. PS training can be brief (i.e., involve fewer than 10 sessions) [38], and has been delivered to youth by trained clinicians [45], lay counsellors [46], and via online platforms [44]. It can also be adapted for primary care [40]. In light of its versatility and of its effectiveness in adults, PS training is a prime candidate for a treatment ingredient that deserves greater scrutiny in the context of youth depression. However, no systematic evidence synthesis has yet examined its efficacy and effectiveness in this population.

This study had two sequential parts. First, we conducted a mixed-methods scoping review to map the available evidence relating to PS training as an active ingredient for treating youth depression. Youth were defined as aged 14 to 24 years, broadly aligning with United Nations definitions [48]. In a subsequent step, we conducted an exploratory meta-analysis to examine the overall efficacy of free-standing PST, based on clinical trials identified in the scoping review.

## Methods

### Scoping review

Scoping review methodology was used to provide an initial overview of the available evidence [49]. The review was pre-registered on the Open Science Framework [50] and adhered to the Preferred Reporting Items for Systematic Reviews and Meta-Analysis (PRISMA) extension for Scoping Reviews checklist [51] (Additional File 1). The review was designed to integrate four types of literature: (a) qualitative studies reporting on young people’s experiences with PS training; (b) controlled clinical trials testing the efficacy of stand-alone PST; (c) studies examining PS-related concepts as predictors, moderators, or mediators of treatment response within broader therapeutic interventions (e.g., CBT); and (d) clinical practice guidelines (CPGs) for youth depression. In addition, the search strategy included terms designed to identify relevant conceptual articles that are discussed here as part of the introduction [52].

#### Search strategy

Five bibliographic databases (APA PsycINFO, CINAHL, Embase, MEDLINE, Web of Science) and the grey literature were systematically searched for (a) empirical studies published from database inception through June 2020, and (b) CPGs published between 2005 and July 2020. Reference lists of key studies were searched manually, and records citing key studies were searched using Google Scholar’s “search within citing articles” function [52]. The search strategy was designed in collaboration with a research librarian (SB) and combined topic-specific terms defining the target population (e.g., “depression”; “adolescent?”) and intervention (e.g., “problem-solving”) with methodological search filters combining database-specific subject headings (e.g., “randomized controlled trial”) and recommended search terms. The search for CPGs built upon a previous systematic search [53, 54], which was updated and expanded to cover additional languages and databases. A multi-pronged grey literature search retrieved records from common grey literature databases and CPG repositories, websites of relevant associations, charities, and government agencies. The search strategy is provided in Additional File 2.

#### Inclusion and exclusion criteria

Empirical studies were included if the mean participant age fell within the eligible range of 14 to 24 years, and at least 50% of participants showed above-threshold depressive or emotional symptoms on a validated screening tool. Controlled clinical trials had to compare the efficacy or effectiveness of PST as a free-standing intervention with a control group or waitlist condition. Secondary analyses were considered for their assessment of PS ability as a predictor, moderator, or mediator of treatment response if they reported on data from controlled clinical trials of broader therapy packages. Records were included as CPGs if labelled as practice guidelines, practice parameters, or consensus or expert committee recommendations, or explicitly aimed to develop original clinical guidance [53, 54]; and if focused on indicated psychosocial treatments for youth depression (rather than prevention, screening, or pharmacological treatment). Doctoral dissertations were included. Conference abstracts, non-controlled trials, and prevention studies were excluded. Language of publication was restricted to English, French, German, and Spanish.

#### Screening

**Fig. 2:**
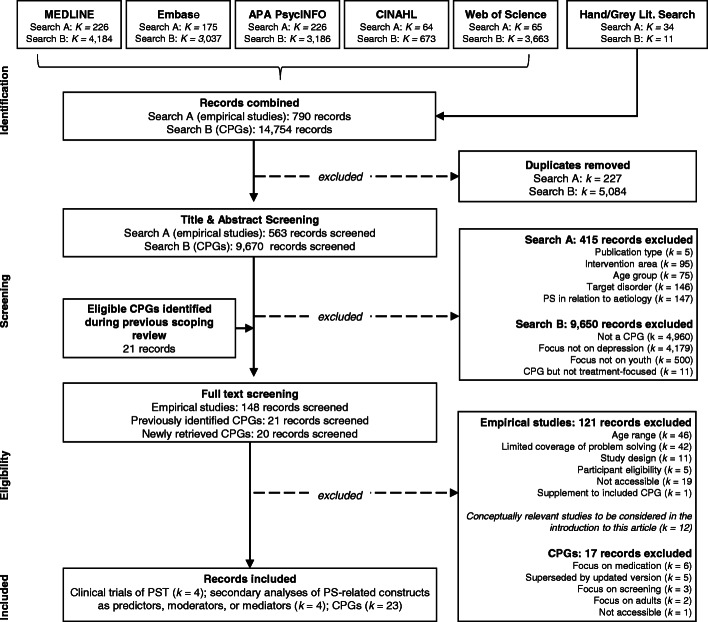
PRISMA Flow Chart of the Study Selection Process

All records identified were imported into the EPPI-Reviewer 4.0 review software [55], and underwent a two-stage screening process (Fig. 2). Title and abstract screening was conducted in duplicate for 10% of the identified records, yielding substantial inter-rater agreement (*kappa* = .75 and .86, for empirical studies and CPGs, respectively). Of studies retained for full text screening, 20% were screened in duplicate, yielding substantial agreement (*kappa* = .68 and .71, for empirical studies and CPGs, respectively). Disagreements were resolved through discussion.

#### Data extraction and synthesis

Data were extracted using templates tailored to each literature type (e.g., the Cochrane data collection form for RCTs). Information extracted included: citation details; study design; participant characteristics; and relevant qualitative or quantitative results. Additional information extracted from CPGs included the issuing authority, the target population, the treatment settings to which the guideline applied, and any recommendations in relation to PS training. Data from clinical trials and secondary analyses were extracted in duplicate, and any discrepancies were discussed and resolved. Data synthesis followed a five-step process of data reduction, display, comparison, conclusion drawing, and verification [56]. Scoping review findings were summarized in narrative format. In addition, effect sizes reported in PST trials for depression severity were entered into an exploratory meta-analysis (see below).

The Centre for Addiction and Mental Health (CAMH) implements a *Youth Engagement Initiative* that brings the voices of youth with lived experience of mental health difficulties into research and service design [57–59]. Two youth partners were co-investigators in this review and consulted with a panel of twelve CAMH youth advisors to inform the review process and help contextualize findings. Formal approval by a Research Ethics Board (REB) was not required, as youth were research partners rather than participants.

To incorporate a variety of perspectives, the review team convened for an inference workshop where emerging review findings and feedback from youth advisors were discussed and interpreted. The multidisciplinary team involved a methodologist; two child and adolescent psychiatrists with expertise in CBT, DBT, and IPT; a psychologist with expertise in parent-adolescent therapy; a research librarian; a family doctor; a biostatistician; a clinical epidemiologist; two youth research partners; and a youth engagement coordinator.

### Exploratory Meta-analysis

Although meta-analyses are not typical components of scoping reviews [60], an exploratory meta-analysis was conducted following completion of the scoping review and narrative synthesis, to obtain an initial indication of the efficacy of stand-alone PST based on the clinical trials identified in the review. The PICO statement that guided the meta-analysis is shown in Table 1.

**Table 1:** PICO Statement Guiding the Exploratory Meta-Analysis

PICO	Description
**P** (Population)	Youth aged 14–24 years (i.e., mean age of the sample within this range) experiencing depression (i.e., in samples with mixed presenting problems, > 50% of youth must have above-threshold symptoms of depression or emotional difficulties).
**I** (Intervention)	Problem-Solving Therapy (i.e., problem-solving training delivered as an intervention in its own right, rather than a component of a broader therapy package).
**C** (Comparison)	Waitlist, placebo, attention placebo, or control condition.
**O** (Outcome)	Self-reported depression severity (continuous).

#### Quality assessment

Risk of bias for included PST trials was appraised using the Cochrane Collaborations Risk of Bias (ROB) 2 tool [61]. Ratings were performed independently by two reviewers (KRK and MA), and consensus was formed through discussion. In addition, a Grading of Recommendations Assessment, Development, and Evaluation (GRADE) appraisal was conducted (using the GRADEpro software; [62] to characterize the quality of the overall evidence. The evidence was graded for risk of bias, imprecision, indirectness, inconsistency, and publication bias [63]. A GRADE of “high quality” indicates a high level of confidence that the true effect lies close to the estimate; “moderate quality” indicates moderate confidence; “low quality” indicates limited confidence; and “very low quality” indicates very little confidence in the estimate. ROB ratings and GRADE appraisal results are provided in Additional File 6.

#### Statistical analysis

The meta-analysis was conducted using the *meta* suite of commands in Stata 16.1. Effect sizes (Hedges’ g) and their confidence intervals were calculated based on the mean difference in depression severity scores between the PST and control conditions at the first post-treatment assessment [64]. Hedges’ g is calculated by subtracting the post-treatment mean score of the intervention group from the score of the control group, and by dividing the mean difference by the pooled standard deviation. Effect sizes between g = 0.2 and 0.5 indicate a small effect; g = 0.5 to 0.8 indicates a moderate effect; and g ≥ 0.8 indicates a large effect. Effect sizes were adjusted using the Hedges and Olkin small sample correction [64]. Pooled effect sizes were computed using a random effects model to account for heterogeneity in intervention settings, modes of delivery, and participant age and depression severity. The *I*^*2*^ statistic was computed as an indicator of effect size heterogeneity. Higgins et al. [65] suggest that an *I*^*2*^ below 30% represents low heterogeneity while an *I*^*2*^ above 75% represents substantial heterogeneity. Investigations of heterogeneity are unlikely to generate valuable insights in small study samples, with at least ten studies recommended for meta-regression [65]. We conducted limited exploratory subgroup analysis by computing a separate effect size after excluding studies with high risk of bias. We inspected the funnel plot and considered conducting Egger’s test to examine the likelihood and extent of publication bias [66].

## Results

### Selection and inclusion of studies

The search for empirical studies identified 563 unique records (Fig. 2), of which 148 were screened in full. Inclusion criteria were met by four RCTs of free-standing PST and four secondary analyses of clinical trials investigating PS-related concepts as predictors, mediators, or moderators of treatment response. No eligible qualitative studies that explicitly examined youth experiences of PS training were identified. The search for CPGs identified 9691 unique records, of which 41 were subject to full text screening, and 23 were included in the review. Below we present scoping review findings for all literature types, followed by the results from the meta-analysis for stand-alone PST trials.

### Clinical trials of PST

Characteristics of the included PST trials are shown in Table 2. Studies were published between 2008 and 2020 and included 524 participants (range: 45 to 251), with a mean age of 16.7 years (range: 12–25; 48% female). Participants had a diagnosis of major depressive disorder (MDD; *k* = 1), elevated anxiety or depressive symptoms (*k* = 1), or various mild presenting problems including depression (*k* = 2). Treatment covered PS skills but not problem orientation (i.e., youth’s problem appraisals) and was delivered face to face (*k =* 3) or online (*k* = 1) in five to six sessions. PST was compared with waitlist controls (*k* = 2), PS booklets (*k =* 1), and supportive counselling (*k* = 1). Risk of bias was rated as medium for two [44, 45], and high for one study [43] due to concerns about missing outcome data and the absence of a study protocol.

**Table 2:** Study Characteristics—Clinical Trials of Stand-Alone PST

Study	Country	*N*^a^	Participant age range (mean) in years	% female	Diagnostic status	% with elevated depressive symptoms	Recruitment Setting	Conditions	*N* sessions	Outcome considered for meta-analysis	Risk of Bias
Eskin et al. [43]	Turkey	53	N/A (19.1)	70	Diag. (Dep)	100	Community	1. PST 2. Waitlist	6	BDI (Prim)	High
Hoek et al. [44]	Netherlands	45	12–21 (16.1)	76	Elev. (Anx or Dep)	80	Community	1. PST 2. Waitlist	5	CES-D (Prim)	Some concerns
Parker et al. [45]	Australia	176	15–25 (17.6)	61	Elev. (various)	54	Clinical	1. PST ^c^ 2. SUP ^c^	6	BDI-II (Prim)	Some concerns
Michelson et al. [46]	India	251	12–20 (15.6)	30	Elev. (various)	53^b^	Community	1. PST 2. PST via booklets only	5	SDQ emotional symptoms (Sec)	Low

Note. BDI: Beck Depression Inventory [67]; CES-D: Center for Epidemiologic Studies Depression Scale [68]; Diag.: Diagnosis; Elev.: elevated symptoms; Prim: defined as a primary outcome in the primary study; SDQ = Strengths and Difficulties Questionnaire [69]; Sec: defined as a secondary outcome in the primary study; SUP: supportive counselling

^a^The total sample size reported is the number of participants randomized to intervention and control conditions

^b^This study assessed broader emotional symptoms, rather than depressive symptoms, via the SDQ’s emotional symptoms subscale

^c^Delivered with adjunctive behavioural activation or psychoeducation in a factorial 2 × 2 design

Eskin and colleagues [43] randomized 53 Turkish high school and university students with MDD to six sessions of PST or a waitlist. The study reports a significant treatment effect on self-reported depressive symptoms (d = − 1.20; F [1, 42] = 10.3, *p* < .01.), clinician-reported depressive symptoms (d = − 2.12; F [1, 42] = 37.7, *p* < .001), and recovery rates, but not on self-reported PS ability (d = − 0.46; F [1, 42] = 2.2, *p* > .05). Risk of bias was rated as high due to 37% of missing outcome data in the control group and the absence of a published trial protocol.

Michelson and colleagues [46] compared PST delivered by lay counsellors in combination with booklets, to PS booklets alone in 251 high-school students with mild mental health difficulties (53% emotional problems) in low-income communities in New Delhi, India. At six weeks, the intervention group showed significantly greater progress towards overcoming idiographic priority problems identified at baseline (d = 0.36, *p* = .002), but no significant difference in self-reported mental health difficulties (d = 0.16, *p* = .18). Results were similar at 12 weeks, including no significant difference in self-reported emotional symptoms (d = 0.18, *p* = .089). As there was no long-term follow-up, it is unknown whether reduced personal problems translated into reduced emotional symptoms in the longer term. Perceived stress at six weeks was found to mediate treatment effect on idiographic problems, accounting for 15% of the overall effect at 12 weeks.

Two trials found no significant effect of PST on primary or secondary outcomes: Hoek and colleagues [44] randomized 45 youth with elevated depression or anxiety symptoms to five sessions of online PST or a waitlist control; Parker and colleagues [45] randomized 176 youth with mixed presenting problems (54% depression) to either PST with physical activity or PST with psychoeducation, compared with supportive counselling with physical activity or psychoeducation [45]. Drop-out from PST was high in both studies, ranging from 41.4% [45] to 72.7% [44].

### PS-related concepts as predictors, moderators, or mediators of treatment response

The review identified four secondary analyses of RCT data that examined PS-related concepts as predictors, moderators, or mediators of treatment response (see Table 3, below). Studies were published between 2005 and 2014 and included data from 761 participants with MDD diagnoses, and a mean age of 15.2 years (range: 12–18; 61.2% female).

**Table 3:** Study Characteristics and Findings—Secondary Analyses Testing PS as a Predictor, Moderator, or Mediator of Treatment Response

Study	Country	*N*	Participant age range (mean) in years	Diagnostic Status	Recruitment Setting	Overarching intervention(s)	Effect type	Operationalization of problem solving	Outcome	Effect
Becker-Weidman et al. [70]	USA	439 ^a^	12–17 (14.6)	MDD	Clinical	1. Fluox 2. CBT 3. Fluox + CBT 4. Placebo	Pred / Mod	Baseline PPO, NPO, AS, ICS, RPS,	Depression severity (CDRS-R)	NPO, PPO, AS = Pred**
Kennard et al. [71]	USA	166 ^b^	12–18 (16.0)	MDD (TR)	Clinical	Switch to: 1. Diff SSRI 2. Diff. SSRI + CBT 3. Ven 4. Ven + CBT	Pred	Receipt of PS training as part of CBT vs. no receipt	Treatment response (CDRS-R & CGI-I)	PS training receipt = Pred*
Kaufman et al. [72]	USA	93 ^a^	13–17 (15.1)	MDD & CD	Community	1. CWD-A 2. LST	Med	Δ self-reported PS in specific situations	Depression severity (BDI-II & HDRS)	No significant effect
Dietz et al. [73]	USA	63 ^a^	13–18 (15.6)	MDD	Clinical	1. CBT 2. SBFT 3. NST	Med	Δ observed interpersonal PS interactions between youth and mothers	MDD remission (K-SADS & BDI).	Δ in PS interactions associated with outcome in CBT** and SFBT* but test for formal mediation not significant

*Note.* Δ: Change in; AS: Avoidance Style; BDI: Beck Depression Inventory [74]; CD: Conduct disorder; CDRS-R: Children’s Depression Rating Scale—Revised [75]; CGI-I: Clinical Global Impression Scale—Improvement [76]; CWD-A: Adolescent Coping with Depression Course; Diff: different; Fluox: Fluoxetine; ICS: Impulsivity/Carelessness Style; K-SADS: The Kiddie Schedule for Affective Disorders and Schizophrenia [77]; LST: Lifeskills training; MDD: Major Depressive Disorder; Med: Mediator; Mod: Moderator; NPO: Negative Problem Orientation; NST: Nondirective supportive therapy; PPO: Positive Problem Orientation; Pred: Predictor; RPS: Rational Problem Solving Style; SBFT: Systemic Behaviour Family Therapy; SPSI-R: Social Problem-Solving Inventory Revised [78]; SSRI: Selective serotonin reuptake inhibitors; TR: Treatment-resistant; Ven: Venlafaxine

* *p* < .05; ** *p* < .01; *** *p* < .001

^a^ The total sample size reported is the number of participants randomized to intervention and control conditions

^b^ The total sample size reported here is the number of participants randomized to an SSRI + CBT intervention arm

A secondary analysis of data from the Treatment for Adolescents with Depression Study (TADS, *n* = 439) [79] explored whether baseline problem orientation and PS styles were significant predictors or moderators of treatment response to Fluoxetine, CBT, or a combination treatment at 12 weeks [70]. Negative problem orientation and avoidant PS style each predicted less improvement in depression symptom severity (*p* = .001 and *p* = .003, respectively), while positive problem orientation predicted greater improvement (*p* = .002). There was no significant moderation effect. Neither rational PS style nor impulsive-careless PS style predicted or moderated change in depressive symptoms.

A secondary analysis of data from the Treatment of Resistant Depression in Adolescents (TORDIA) study [80] examined the impact of specific CBT components on treatment response at 12 weeks in youth treated with a selective serotonin reuptake inhibitor (SSRI) in combination with CBT (*n* = 166) [71]. Youth who received PS training were 2.3 times (*p* = .03) more likely to have a positive treatment response than those not receiving this component. A significant effect was also observed for social skills training (Odds Ratio [OR] = 2.6, *p* = .04) but not for seven other CBT components. PS and social skills training had the most equal allocation ratios between youth who received them (52 and 54%, respectively) and youth who did not. Balanced allocation provides maximum power for a given sample size [81]. With allocation ratios between 1:3 and 1:5, analysis of the remaining seven components may have been underpowered. Of further note, CBT components were not randomly assigned but selected based on individual clinical needs. The authors did not correct for multiple comparisons as part of this exploratory analysis.

Dietz and colleagues [73] explored the impact of social problem solving on treatment outcome based on data from a trial comparing CBT and Systemic Behaviour Family Therapy (SBFT) with elements of PS training on the one hand, with Non-Directive Supportive Therapy on the other hand (*n* = 63). Both CBT and SBFT were associated with significant improvements in young people’s interpersonal PS behaviour (measured by coding videotaped interactions between youth and their mothers) over the course of treatment (CBT: b* = 0.41, *p* = .006; SBFT: b* = 0.30, *p* = .04), which in turn were associated with higher rates of remission (Wald z = 6.11, *p* = .01). However, there was no significant indirect effect of treatment condition via youth PS behaviour, and hence, no definitive evidence of a formal mediation effect [82].

Kaufman and colleagues [72] examined data from a trial comparing an Adolescent Coping with Depression (CWD-A) group-based intervention with a life-skills control condition in 93 youth with comorbid depression and conduct disorder. The secondary analysis explored whether change in six CBT-specific factors, including the use of PS and conflict resolution skills, mediated the effectiveness of CWD-A. There was no significant improvement in PS ability in CWD-A, compared with the control, and hence no further mediation analysis was conducted.

### PS training in clinical practice guidelines

We identified 23 CPGs from twelve countries relevant to youth depression (see Additional File 4), issued by governments (*k* = 6), specialty societies (*k* = 3), health care providers (*k* = 4), independent expert groups (*k* = 2), and others, or a combination of these. Of these 23 CPGs, 15 mentioned PS training in relation to depression treatment for youth, as a component of CBT (*k* = 7), IPT (*k* = 4), supportive therapy or counselling (*k* = 3), family therapy (*k* = 1), DBT (*k* = 1), and psychoeducation (*k* = 1).

None of the reviewed CPGs recommended free-standing PST as a first-line treatment for youth depression. However, five CPGs mentioned PS training as a treatment ingredient or adjunct component in the context of recommending broader therapeutic approaches. The World Health Organization’s updated Mental Health Gap Action Programme guidelines recommended PS training as an adjunct treatment (e.g., in combination with antidepressant medication) for older adolescents [83]. A guideline by Orygen (Australia) suggested that for “persistent sub-threshold depressive symptoms (including dysthymia) or mild to moderate depression”, options should include “6–8 sessions of individual guided self-help based on the principles of CBT, including behavioural activation and problem-solving techniques” [84]. The Chilean Ministry of Health recommended supportive clinical care with adjunctive psychoeducation and PS tools, or supportive counselling for individuals aged 15 and older with mild depression (p. 52) [85]. The Cincinnati Children’s Hospital Medical Centre recommended four to eight sessions of supportive therapy for mild or uncomplicated depression, highlighting “problem solving coping skills” as one element of supportive therapy (p. 1) [86]. Fifth, the American Academy of Child and Adolescent Psychiatry’s 2007 practice parameter suggested each phase of treatment for youth depression should include psychoeducation and supportive management, which might include PS training (p. 1510) [87]. CPGs did not specify whether PS training should incorporate specific modules, or whether the term was used loosely to describe unstructured PS support.

### Meta-analysis

**Fig. 3:**
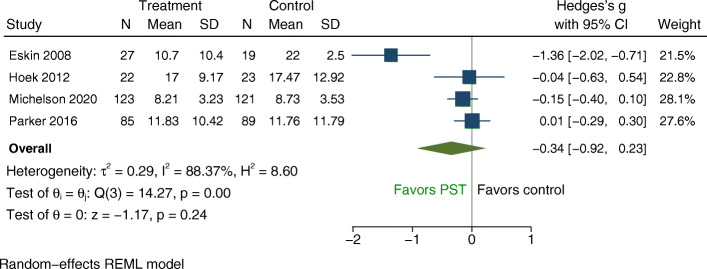
Forest Plot: Random Effects Model with Self-Reported Depression or Emotional Symptoms as Primary Outcome (Continuous)

Each of the four RCTs of free-standing PST identified by the scoping review contributed one comparison to the exploratory meta-analysis of overall PST efficacy (see Fig. 3). Self-rated depression or emotional symptom severity scores were reported by all four studies and constituted the primary outcome for the meta-analysis. We conducted additional exploratory analysis for clinician-rated depression severity as reported in two studies [43, 45]. The pooled effect size for self-reported depression severity was g = − 0.34 (95% CI: − 0.92 to 0.23). Heterogeneity was high (*I*^*2*^ = 88.37%; *p* < .001). Due to the small number of studies included, analysis of publication bias via an examination of the funnel plot and tests of funnel plot asymmetry could not be meaningfully conducted [88, 89]. The funnel plot is provided in Additional File 5 for reference (Fig. S3).

To achieve the best possible estimate of the true effect size and reduce heterogeneity we computed a second model excluding the one study with high risk of bias (i.e., [43]). The resulting effect size was g = − 0.08 (95% CI: − 0.26 to 0.10), with no significant heterogeneity (*I*^*2*^ = 0.00%; *p* = 0.72; see Fig. S1 in Additional File 5). The pooled effect size for clinician-rated depression severity was g = − 1.39 with a wide confidence interval (95% CI: − 4.03 to 1.42) and very high heterogeneity (*I*^2^ = 97.41%, *p* < 0.001; see Fig. S2 in Additional File 5).

#### Overall quality of the evidence

According to the GRADE assessment, the overall quality of the evidence was very low, with concerns related to risk of bias, the inconsistency of results across studies, the indirectness of the evidence with regards to the population of interest (i.e., only one trial focused exclusively on youth with depression), and imprecision in the effect estimate (Table S4 in Additional File 6).

## Discussion

This scoping review aimed to provide a first comprehensive overview of the evidence relating to PS training as an active ingredient for treating youth depression. The evidence base relating to the efficacy of PST as a stand-alone intervention was scarce and of low quality. Overall, data from four trials suggested no significant effect on depression symptoms. The scoping review identified some evidence suggesting PS training may enhance treatment response in CBT. However, this conclusion was drawn from secondary analyses where youth were not randomized to treatment with and without PS training, and where primary studies were not powered to test these differences. Disproportionate exposure to comparator CBT components also limits these findings. PST was not recommended as a stand-alone treatment for youth depression in any of the 23 reviewed CPGs; however, one guideline suggested it could be provided alongside other treatments for older adolescents, and four suggested PS training as a component of low-intensity psychosocial interventions for youth with mild to moderate depression.

Given the limited evidence base, only tentative suggestions can be made as to when and for whom PS training is effective. The one PST trial with a low risk of bias enrolled high-school students from low-income communities in New Delhi, and found that PST delivered by lay counselors in combination with PST booklets was more effective at reducing idiographic priority problems than booklets alone, but not at reducing mental health symptoms [46]. Within a needs-based framework of service delivery (e.g., [90]), PST may be offered as a low-intensity intervention to youth who experience challenges *and* struggle with PS—including in low-resource contexts. Future research could explore whether PS training might be particularly helpful for youth facing socioeconomic hardship and related chronic stressors by attenuating potentially harmful impacts on well-being [91]. If findings are promising, PS training may be considered for targeted prevention (e.g., [42]). However, at this time there is insufficient evidence to support PS training on its own as an intervention aimed at providing symptom relief for youth experiencing depression.

The PST manual suggests cognitive overload, emotional dysregulation, negative thinking and hopelessness can interfere with PS [16]. Youth whose depression hinders their ability to engage in PST may require additional support through more comprehensive therapy packages such as CBT or IPT with PS training. In the TORDIA study [80], where PS training was found to be one of the most effective components, it was generally taught alongside cognitive restructuring, behavioural activation, and emotion regulation, which may have facilitated youths’ ability to absorb PS training [71]. The focus of these other CBT components on changing negative cognitions and attributions may fulfil a similar function as problem orientation modules in stand-alone PST. Research that is powered to explore such mechanisms is needed. Future research should also apply methodologies designed to identify the most critical elements in a larger treatment package (e.g., dismantling studies; or sequential, multiple assignment, randomized trials) to examine the role of PS training when delivered alongside other components. While one trial focusing on CBT components is currently underway [92], similar research is needed for other therapies (e.g., IPT, DBT, family therapy).

The included PST trials provided between five and six sessions and covered PS skills but not problem orientation. Meta-analyses of PST for adult depression suggest treatment effectiveness may be enhanced by longer treatment duration (≥ 10 sessions) [38], and coverage of problem orientation alongside PS skills [39]. As per the PST treatment manual, strengthening problem orientation fosters motivation and self-efficacy and is an important precondition for enhancing skills [93, 94]. In addition, only one youth PST trial assessed PS ability at baseline [43]. A meta-analysis of PST for adult depression [39] suggests that studies including such assessments show larger effect sizes, with therapists better able to tailor PST to individual needs. Future research should seek to replicate these findings specifically for youth depression.

Drop out from stand-alone PST was high in two out of four studies, ranging from 41.4% [45] to 72.7% [44]. Since its development in the 1970s, PST has undergone several revisions [16, 93, 95–97] but tailoring to youth has been limited. To contextualize the review findings, the review team consulted a panel of twelve youth advisors at the Centre for Addiction and Mental Health (without sharing emerging findings so as not to steer the conversation). Most had participated in PS training as part of other therapies, but none had received formal PST. A key challenge identified by youth advisors was how to provide PS training that is universally applicable and relevant to different youth without being too generic, rigid or schematic; and how to accommodate youth perspectives, complex problems, and individual situations and dispositions. Youth advisors suggested reviewing and reworking PS training with youth in mind, to ensure it is youth-driven, strengths-based, comprehensive, and personalized (see Fig. S4 in Additional File 7 for more detail). Youth advisors emphasized that PS training should identify the root causes underpinning superficial problems and address these through suitable complementary intervention approaches, if needed.

Solution-focused brief therapy (SFBT) has emerged as an antithesis to PST where more emphasis is given to envisaging and constructing solutions rather than analysing problems [28]. This may be more consistent with youth preferences for strengths-based approaches but may provide insufficiently comprehensive problem appraisals. Future research should compare the effectiveness and acceptability of PST and SFBT and consider possible benefits of combining the advantages of both approaches, to provide support that is strengths-based and targets root problems. More generally, given the effectiveness of PST in adults, future studies could examine whether there are developmental factors that might contribute to reduced effectiveness in youth and should be considered when adapting PST to this age group.

### Strengths and limitations

This scoping review applied a broad and systematic approach to study identification and selection. We searched five bibliographic databases, and conducted an extensive grey literature search, considering records published in four languages. Nevertheless, our search may have missed relevant studies published in other languages. We found only a small number of eligible empirical studies, several of which were likely underpowered. As stated above, studies analysing PS-related concepts as predictors, moderators, or mediators of treatment response within broader therapies were heterogenous and limited by design and sample size constraints.

Similarly, there was heterogeneity in recruitment and intervention settings, age groups, and delivery formats across the four RCTs of stand-alone PST, and the overall quality of the evidence was very low. As reflected in our GRADE appraisal, one important limitation was the indirectness of the available evidence: Only one PST trial focused specifically on youth with an MDD diagnosis, while the remaining three included youth with a mix of mental health problems. Although outcomes were reported in terms of depression or emotional symptom severity, this was not based on a subgroup analysis focused specifically on youth with depression. Impact on this group may therefore have been underestimated. In addition, the only PST trial with a low risk of bias did not administer a dedicated depression symptom scale. Instead, our exploratory meta-analysis included scores from the 5-item SDQ emotional problems subscale, which assesses unhappiness, worries, clinginess, fears, and somatic symptoms—and may not have captured nuanced change in depression severity [98, 99]. Other concerns that led us to downgrade the quality of the evidence related to considerable risk of bias, with only one out of four studies rated as having a low risk; and imprecision with several studies involving very small samples. Due to the small number of eligible studies, it was not possible to identify the factors driving treatment efficacy via meta-regression. The long-term effectiveness of PS training, or the conditions under which long-term benefits are likely to be realized also could not be examined [38].

## Conclusions

PS training is a core component of several evidence-based therapies for youth depression. However, the evidence base supporting its efficacy as a stand-alone treatment is limited and of low quality. There is tentative evidence suggesting PS-training may drive positive outcomes when provided alongside other treatment components. On its own, PS training may be beneficial for youth who are not acutely distressed or impaired but require support with tackling personal problems. Youth experiencing moderate or severe depressive symptoms may require more comprehensive psychotherapeutic support alongside PS training, as there is currently no robust evidence for the ability of free-standing PST to effectively reduce depression symptoms.

High-quality trials are needed that assess PST efficacy in youth with mild, moderate, and severe depression, in relation to both symptom severity and idiographic treatment goals or priority problems. These studies should examine the influence of treatment length and module content on treatment impact. Dedicated studies are also needed to shed light on the role of PS training as an active ingredient of more comprehensive therapies such as CBT, DBT, IPT, and family therapy. Future studies should include assessments of adverse events and of cost effectiveness. Given high drop-out rates in several youth PST trials, it is important to adapt PS training approaches and therapy manuals as needed, following a youth-engaged research and service development approach [57], to ensure their relevance and acceptability to this age group.

## Supplementary Information

**Additional file 1.** Preferred Reporting Items for Systematic reviews and Meta-Analyses extension for Scoping Reviews (PRISMA-ScR) Checklist.

**Additional file 2.** Search Strategy.

**Additional file 3.** List of Studies Included in the Scoping Review.

**Additional file 4.** Characteristics of Included Clinical Practice Guidelines.

**Additional file 5.** Additional Data and Outputs from the Meta-Analysis.

**Additional file 6.** Risk of Bias Assessment and GRADE Appraisal.

**Additional file 7.** Illustration of Insights from the Consultation of Youth Advisors.

## Data Availability

All data generated or analysed during this study are included in this published article and its supplementary information files.

## Abbreviations

AS: Avoidance style
BDI: Beck Depression Inventory
CAMH: Centre for Addiction and Mental Health
CBT: Cognitive behavioural therapy
CDRS-R: Children’s Depression Rating Scale—Revised
CES-D: Center for Epidemiologic Studies Depression Scale
CGI-I: Clinical Global Impression Scale—Improvement
CINAHL: Cumulative Index to Nursing and Allied Health Literature
CPG: Clinical practice guideline
CWD-A: Adolescent Coping with Depression [intervention name]
DBT: Dialectical behaviour therapy
GRADE: Grading of Recommendations Assessment, Development, and Evaluation
ICS: Impulsivity/Carelessness Style
IPT: Interpersonal psychotherapy
K-SADS: The Kiddie Schedule for Affective Disorders and Schizophrenia
LST: Lifeskills training
MDD: Major depressive disorder
Medline: Medical Literature Analysis and Retrieval System Online
NPO: Negative problem orientation
NST: Nondirective supportive therapy
OR: Odds ratio
PPO: Positive problem orientation
PRISMA: Preferred Reporting Items for Systematic Reviews and Meta-Analysis
PS: Problem solving
PS Training: Problem-solving training
PST: Problem-Solving Therapy
RCT: Randomized controlled trial
REB: Research ethics board
ROB: Risk of bias
RPS: Rational problem-solving style
SBFT: Systemic Behaviour Family Therapy
SDQ: Strengths and Difficulties Questionnaire
SFBT: Solution-Focused Brief Therapy
SPSI-R: Social Problem-Solving Inventory Revised
SSRI: Selective serotonin reuptake inhibitors
TADS: Treatment for Adolescents with Depression Study
TORDIA: Treatment of Resistant Depression in Adolescents

## References

[1] Polanczyk GV, Salum GA, Sugaya LS, Caye A, Rohde LA (2015). Annual research review: a meta-analysis of the worldwide prevalence of mental disorders in children and adolescents. J Child Psychol Psychiatry.

[2] Avenevoli S, Swendsen J, He J-P, Burstein M, Merikangas KR (2015). Major Depression in the National Comorbidity Survey–Adolescent Supplement: Prevalence, Correlates, and Treatment. J Am Acad Child Adolesc Psychiatry.

[3] Gore FM, Bloem PJN, Patton GC, Ferguson J, Joseph V, Coffey C, Sawyer SM, Mathers CD (2011). Global burden of disease in young people aged 10–24 years: a systematic analysis. Lancet..

[4] Fergusson DM, Woodward LJ (2002). Mental health, educational, and social role outcomes of adolescents with depression. Arch Gen Psychiatry.

[5] Jonsson U, Bohman H, von Knorring L, Olsson G, Paaren A, von Knorring A-L (2011). Mental health outcome of long-term and episodic adolescent depression: 15-year follow-up of a community sample. J Affect Disord.

[6] Clayborne ZM, Varin M, Colman I (2019). Systematic review and Meta-analysis: adolescent depression and long-term psychosocial outcomes. J Am Acad Child Adolesc Psychiatry.

[7] Fletcher JM (2008). Adolescent depression: diagnosis, treatment, and educational attainment. Health Econ.

[8] Fletcher JM (2013). Adolescent depression and adult labor market outcomes. South Econ J.

[9] Zhou X, Hetrick SE, Cuijpers P, Qin B, Barth J, Whittington CJ, Cohen D, del Giovane C, Liu Y, Michael KD, Zhang Y, Weisz JR, Xie P (2015). Comparative efficacy and acceptability of psychotherapies for depression in children and adolescents: a systematic review and network meta-analysis. World Psychiatry.

[10] Zhou X, Teng T, Zhang Y, Del Giovane C, Furukawa TA, Weisz JR (2020). Comparative efficacy and acceptability of antidepressants, psychotherapies, and their combination for acute treatment of children and adolescents with depressive disorder: a systematic review and network meta-analysis. Lancet Psychiatry.

[11] Bear HA, Edbrooke-Childs J, Norton S, Krause KR, Wolpert M (2020). Systematic review and Meta-analysis: outcomes of routine specialist mental health Care for Young People with Depression and/or anxiety. J Am Acad Child Adolesc Psychiatry.

[12] Cuijpers P, Stringaris A, Wolpert M (2020). Treatment outcomes for depression: challenges and opportunities. Lancet Psychiatry.

[13] Kazdin AE (2009). Understanding how and why psychotherapy leads to change. Psychother Res.

[14] de Haan AM, Boon AE, de Jong JTVM, Hoeve M, Vermeiren RRJM (2013). A meta-analytic review on treatment dropout in child and adolescent outpatient mental health care. Clin Psychol Rev.

[15] Chorpita BF, Daleiden EL (2009). Mapping evidence-based treatments for children and adolescents: application of the distillation and matching model to 615 treatments from 322 randomized trials. J Consult Clin Psychol.

[16] Nezu AM, Nezu CM, D’Zurilla TJ. Problem-solving therapy: A treatment manual. Problem-solving therapy: A treatment manual: Springer Publishing Company; 2013. x, 323–x, 323

[17] D’Zurilla TJ, Nezu AM (2010). Problem-solving therapy. In: Handbook of Cognitive-Behavioral Therapies. Third.

[18] Nezu AM (2004). Problem solving and behavior therapy revisited. Behav Ther.

[19] D’Zurilla TJ, Nezu AM, Maydeu-Olivares A. Social Problem Solving: Theory and Assessment. In: Chang EC, D’Zurilla TJ, Sanna LJ, editors. Social problem solving: Theory, research, and training: American Psychological Association; 2004. p. 11–27.

[20] Ugueto AM, Santucci LC, Krumholz LS, Weisz JR, Sburlati ES, Lyneham HJ, Schniering CA, Rapee RM (2014). Problem-Solving Skills Training. Evidence-based CBT for anxiety and depression in children and adolescents: a competencies-based approach.

[21] D’Zurilla TJ, Chang EC, Nottingham EJ IV, Faccini L. Social problem-solving deficits and hopelessness, depression, and suicidal risk in college students and psychiatric inpatients. J Clin Psychol. 1998;54(8):1091–107. 10.1002/(SICI)1097-4679(199812)54:8<1091::AID-JCLP9>3.0.CO;2-J. 10.1002/(sici)1097-4679(199812)54:8<1091::aid-jclp9>3.0.co;2-j 9840781

[22] Haugh JA (2006). Specificity and social problem-solving: relation to depressive and anxious symptomology. J Soc Clin Psychol.

[23] Nezu AM. Cognitive appraisal of problem solving effectiveness: relation to depression and depressive symptoms. J Clin Psychol. 1986;42(1):42–8. 10.1002/1097-4679(198601)42:1<42::AID-JCLP2270420106>3.0.CO;2-2. 10.1002/1097-4679(198601)42:1<42::aid-jclp2270420106>3.0.co;2-2 3950013

[24] Reinecke MA, Dubois DL, Schultz TM (2001). Social problem solving, mood, and suicidality among inpatient adolescents. Cognit Ther Res.

[25] Siu AMH, Shek DTL (2010). Social problem solving as a predictor of well-being in adolescents and young adults. Soc Indic Res.

[26] D’Zurilla TJ, Goldfried MR (1971). Problem solving and behavior modification. J Abnorm Psychol.

[27] Bandura A (1977). Social learning theory.

[28] Gingerich WJ, Eisengart S (2000). Solution-focused brief therapy: a review of the outcome research. Fam Process.

[29] Boyd RC, Lewis J, Borreggine K, Benton TD (2018). Adolescent depression: identification and treatment. Curr Treat Options Pediatr.

[30] Hamrin V, Pachler MC (2005). Child & adolescent depression: review of the latest evidence-based treatments. J Psychosoc Nurs Ment Health Serv.

[31] McCarty CA, Weisz JR (2007). Effects of psychotherapy for depression in children and adolescents: what we can (and can’t) learn from meta-analysis and component profiling. J Am Acad Child Adolesc Psychiatry.

[32] Fréchette-Simard C, Plante I, Bluteau J (2018). Strategies included in cognitive behavioral therapy programs to treat internalized disorders: a systematic review. Cogn Behav Ther.

[33] Verdeli H, Mufson L, Lee L, Keith J (2006). Review of evidence-based psychotherapies for pediatric mood and anxiety disorders. Curr Psychiatr Rev.

[34] Mufson L, Weissman MM, Moreau D, Garfinkel R (1999). Efficacy of interpersonal psychotherapy for depressed adolescents. Arch Gen Psychiatry.

[35] Diamond G, Siqueland L (1995). Family therapy for the treatment of depressed adolescents. Psychotherapy..

[36] Hallford DJ, Mellor D (2013). Cognitive-reminiscence therapy and usual care for depression in young adults: study protocol for a randomized controlled trial. Trials..

[37] Richardson ED (1998). Adventure-based therapy and self-efficacy theory: Test of a treatment model for late adolescents with depressive symptomatology.

[38] Cuijpers P, de Wit L, Kleiboer A, Karyotaki E, Ebert DD (2018). Problem-solving therapy for adult depression: an updated meta-analysis. Eur Psychiatry.

[39] Bell AC, D’Zurilla TJ (2009). Problem-solving therapy for depression: a meta-analysis. Clin Psychol Rev.

[40] Zhang A, Park S, Sullivan JE, Jing S (2018). The effectiveness of problem-solving therapy for primary care patients’ depressive and/or anxiety disorders: a systematic review and meta-analysis. J Am Board Fam Med.

[41] Stark KD, Reynolds WM, Kaslow NJ (1987). A comparison of the relative efficacy of self-control therapy and a behavioral problem-solving therapy for depression in children. J Abnorm Child Psychol.

[42] Spence SH, Sheffield JK, Donovan CL (2003). Preventing adolescent depression: an evaluation of the problem solving for life program. J Consult Clin Psychol.

[43] Eskin M, Ertekin K, Demir H (2008). Efficacy of a problem-solving therapy for depression and suicide potential in adolescents and young adults. Cognit Ther Res..

[44] Hoek W, Schuurmans J, Koot HM, Cuijpers P (2012). Effects of internet-based guided self-help problem-solving therapy for adolescents with depression and anxiety: a randomized controlled trial. PLoS One.

[45] Parker AG, Hetrick SE, Jorm AF, Mackinnon AJ, McGorry PD, Yung AR (2016). The effectiveness of simple psychological and physical activity interventions for high prevalence mental health problems in young people: a factorial randomised controlled trial. J Affect Disord.

[46] Michelson D, Malik K, Parikh R, Weiss HA, Doyle AM, Bhat B, Sahu R, Chilhate B, Mathur S, Krishna M, Sharma R, Sudhir P, King M, Cuijpers P, Chorpita B, Fairburn CG, Patel V (2020). Effectiveness of a brief lay counsellor-delivered, problem-solving intervention for adolescent mental health problems in urban, low-income schools in India: a randomised controlled trial. Lancet Child Adolesc Heal.

[47] Ng MY, Eckshtain D, Weisz JR (2016). Assessing fit between evidence-based psychotherapies for youth depression and real-life coping in early adolescence. J Clin Child Adolesc Psychol.

[48] United Nations. Youth [Internet]. 2020 [cited 2020 Nov 4]. Available from: https://www.un.org/esa/socdev/documents/youth/fact-sheets/youth-definition.pdf.

[49] Munn Z, Peters MDJ, Stern C, Tufanaru C, McArthur A, Aromataris E (2018). Systematic review or scoping review? Guidance for authors when choosing between a systematic or scoping review approach. BMC Med Res Methodol.

[50] Krause KR, Courtney DB, Bonato S, Aitken M, Hawke LD, Szatmari P. Problem Solving as an Active Ingredient of Treatment for Youth Depression: A Scoping Review [Protocol] [Internet]. [cited 2021 Jan 13]. Available from: osf.io/ydxge 10.1186/s12888-021-03260-9 PMC8383463 34425770

[51] Tricco AC, Lillie E, Zarin W, O’Brien KK, Colquhoun H, Levac D (2018). PRISMA extension for scoping reviews (PRISMA-ScR): checklist and explanation. Ann Intern Med.

[52] Booth A, Carroll C (2015). Systematic searching for theory to inform systematic reviews: is it feasible? Is it desirable?. Health Inf Libr J.

[53] Courtney DB, Duda S, Szatmari P, Henderson J, Bennett K (2019). Systematic review and quality appraisal of practice guidelines for self-harm in children and adolescents. Suicide Life-Threatening Behav.

[54] Bennett K, Duda S, Brouwers M, et al Towards high-quality, useful practice guidelines for child and youth mental health disorders: protocol for a systematic review and consensus exercise BMJ Open. 2018;8:e018053. 10.1136/bmjopen-2017-018053. 10.1136/bmjopen-2017-018053 PMC5829788 29437752

[55] Thomas J, Brunton J, Graziosi S (2010). EPPI-Reviewer 4.0: software for research synthesis.

[56] Whittemore R, Knafl K (2005). The integrative review: updated methodology. J Adv Nurs.

[57] Heffernan OS, Herzog TM, Schiralli JE, Hawke LD, Chaim G, Henderson JL (2017). Implementation of a youth-adult partnership model in youth mental health systems research: challenges and successes. Health Expect.

[58] Hawke LD, Darnay K, Relihan J, Khaleghi-Moghaddam M, Barbic S, Lachance L, Ben-David S, Brown M, Iyer S, Chaim G, Soklaridis S, Kidd SA, Halsall T, Mathias S, Henderson J (2020). Enhancing researcher capacity to engage youth in research: researchers’ engagement experiences, barriers and capacity development priorities. Health Expect.

[59] Hawke LD, Relihan J, Miller J, McCann E, Rong J, Darnay K, Docherty S, Chaim G, Henderson JL (2018). Engaging youth in research planning, design and execution: practical recommendations for researchers. Health Expect.

[60] Peters M, Godfrey C, McInerney P, Munn Z, Trico A, Khalil H. Chapter 11: Scoping Reviews. In: JBI Manual for Evidence Synthesis [Internet]. JBI; 2020. Available from: https://wiki.jbi.global/display/MANUAL/Chapter+11%3A+Scoping+reviews. Accessed 20 Dec 2020.

[61] Sterne JAC, Savović J, Page MJ, Elbers RG, Blencowe NS, Boutron I (2019). RoB 2: a revised tool for assessing risk of bias in randomised trials. BMJ..

[62] GRADEpro GDT. GRADEpro Guideline Development Tool [Software] [Internet]. McMaster University (developed by Evidence Prime, Inc.); 2020. Available from: gradepro.org. Accessed 20 Dec 2020.

[63] Guyatt GH, Oxman AD, Vist GE, Kunz R, Falck-Ytter Y, Alonso-Coello P, Schünemann HJ, GRADE Working Group (2008). GRADE: an emerging consensus on rating quality of evidence and strength of recommendations. BMJ..

[64] Hedges LV (1983). A random effects model for effect sizes. Psychol Bull.

[65] Higgins JPT, Thomas J, Chandler J, Cumpston M, Li T, Page MJ (2019). Cochrane handbook for systematic reviews of interventions.

[66] Egger M, Smith GD, Schneider M, Minder C (1997). Bias in meta-analysis detected by a simple, graphical test. BMJ..

[67] Beck AT, Steer RA. Internal consistencies of the original and revised beck depression inventory. J Clin Psychol. 1984;40(6):1365–7. 10.1002/1097-4679(198411)40:6<1365::AID-JCLP2270400615>3.0.CO;2-D. 10.1002/1097-4679(198411)40:6<1365::aid-jclp2270400615>3.0.co;2-d 6511949

[68] Radloff LS (1977). The CES-D scale. Appl Psychol Meas.

[69] Goodman R (2001). Psychometric properties of the strengths and difficulties questionnaire (SDQ). J Am Acad Child Adolesc Psychiatry.

[70] Becker-Weidman EG, Jacobs RH, Reinecke MA, Silva SG, March JS (2010). Social problem-solving among adolescents treated for depression. Behav Res Ther.

[71] Kennard BD, Clarke GN, Weersing VR, Asarnow JR, Shamseddeen W, Porta G, Berk M, Hughes JL, Spirito A, Emslie GJ, Keller MB, Wagner KD, Brent DA (2009). Effective components of TORDIA cognitive–behavioral therapy for adolescent depression: preliminary findings. J Consult Clin Psychol.

[72] Kaufman NK, Rohde P, Seeley JR, Clarke GN, Stice E (2005). Potential mediators of cognitive-behavioral therapy for adolescents with comorbid major depression and conduct disorder. J Consult Clin Psychol.

[73] Dietz LJ, Marshal MP, Burton CM, Bridge JA, Birmaher B, Kolko D, Duffy JN, Brent DA (2014). Social problem solving among depressed adolescents is enhanced by structured psychotherapies. J Consult Clin Psychol.

[74] Beck AT, Steer RA, Brown GK (1996). Beck depression inventory-ii.

[75] Poznanski E, Mokros H (1996). Children’s depression rating scale–revised (CDRS-R).

[76] Busner J, Targum SD (2007). The clinical global impressions scale: applying a research tool in clinical practice. Psychiatry (Edgmont).

[77] Kaufman J, Birmaher B, Brent D, Rao U, Flynn C, Moreci P (1997). Schedule for affective disorders and schizophrenia for school-age children-present and lifetime version (K-SADS-PL): initial reliability and validity data. J Am Acad Child Adolesc Psychiatry.

[78] D’Zurilla TJ, Nezu AM (2002). Maydeu- Olivares a. social problem-solving inventory - revised: technical manual.

[79] March JS, Silva S, Petrycki S, Curry J, Wells K, Fairbank J, Burns B, Domino M, McNulty S, Vitiello B, Severe J (2007). The treatment for adolescents with depression study (TADS). Arch Gen Psychiatry.

[80] Brent D, Emslie G, Clarke G, Wagner KD, Asarnow JR, Keller M, Vitiello B, Ritz L, Iyengar S, Abebe K, Birmaher B, Ryan N, Kennard B, Hughes C, DeBar L, McCracken J, Strober M, Suddath R, Spirito A, Leonard H, Melhem N, Porta G, Onorato M, Zelazny J (2008). Switching to another SSRI or to venlafaxine with or without cognitive behavioral therapy for adolescents with SSRI-resistant depression. JAMA..

[81] Brysbaert M (2019). How many participants do we have to include in properly powered experiments? A tutorial of power analysis with some simple guidelines. J Cogn.

[82] MacKinnon DP, Warsi G, Dwyer JH (1995). A simulation study of mediated effect measures. Multivariate Behav Res.

[83] World Health Organization. Update of the Mental Health Gap Action Programme (mhGAP) guidelines for mental, neurological and substance use disorders [Internet]. 2015. Available from: https://www.who.int/maternal_child_adolescent/documents/health-promotion-interventions/en/. Accessed 20 Dec 2020. 26937539

[84] Orygen. Treating depression in young people: Guidance, resources and tools for assessment and management [Internet]. Orygen. 2017. Available from: https://www.orygen.org.au/Training/Resources/Depression/Clinical-practice-points/Treating-depression-in-yp. Accessed 20 Dec 2020.

[85] Ministerio de Salud. Guía Clínica Depresión en personas de 15 años y más [Internet]. Santiago, Chile; 2013. Available from: https://www.minsal.cl/portal/url/item/7222754637c08646e04001011f014e64.pdf. Accessed 20 Dec 2020.f

[86] Cincinnati Children's Hospital Medical Center. Best Evidence Statement (BESt): Treatment of Children and Adolescents With Major Depressive Disorder (MDD) During the Acute Phase. Cincinnati: Cincinnati Children's Hospital Medical Center; 2010.

[87] Birmaher B (2007). Practice parameter for the assessment and treatment of children and adolescents with depressive disorders. J Am Acad Child Adolesc Psychiatry.

[88] Lau J, Ioannidis JPA, Terrin N, Schmid CH, Olkin I (2006). The case of the misleading funnel plot. Br Med J.

[89] Sterne JAC, Sutton AJ, Ioannidis JPA, Terrin N, Jones DR, Lau J (2011). Recommendations for examining and interpreting funnel plot asymmetry in meta-analyses of randomised controlled trials. BMJ..

[90] Wolpert M, Harris R, Hodges S, Fuggle P, James R, Wiener A (2019). THRIVE framework for system change.

[91] Hostinar CE, Miller GE (2019). Protective factors for youth confronting economic hardship: current challenges and future avenues in resilience research. Am Psychol.

[92] Van Den Heuvel MWH, Bodden DHM, Moerbeek M, Smit F, Engels RCME (2019). Dismantling the relative effectiveness of core components of cognitive behavioural therapy in preventing depression in adolescents: protocol of a cluster randomized microtrial. BMC Psychiatry.

[93] Nezu AM, Perri MG (1989). Social problem-solving therapy for unipolar depression: an initial dismantling investigation. J Consult Clin Psychol.

[94] D’Zurilla TJ, Nezu AM (2007). Problem-solving therapy: a positive approach to clinical intervention.

[95] Chang EC, D’Zurilla TJ, Sanna LJ. Social problem solving: theory, research, and training: American Psychological Association; 2004. 10.1037/10805-000.

[96] Nezu AM, Nezu CM, Perri MG (1989). Problem-solving therapy for depression: theory, research, and clinical guidelines.

[97] Nezu AM (1987). A problem-solving formulation of depression: a literature review and proposal of a pluralistic model. Clin Psychol Rev.

[98] Goodman R, Meltzer H, Bailey V (1998). The strengths and difficulties questionnaire: a pilot study on the validity of the self-report version. Eur Child Adolesc Psychiatry.

[99] Goodman R (1997). The strengths and difficulties questionnaire: a research note. J Child Psychol Psychiat.

