# Supplementary material for: Problem-solving training as an active ingredient of treatment for youth depression: a scoping review and exploratory meta-analysis
Source: BMC Psychiatry. 2021 Aug 24;21:397. doi: 10.1186/s12888-021-03260-9 (PMC8383463; doi:10.1186/s12888-021-03260-9)
Supplement: Supplementary file 2 — Additional file 2. Search Strategy. [file 12888_2021_3260_MOESM2_ESM.docx]

Problem-Solving Training as an Active Ingredient of Treatment for Youth Depression: A Scoping Review and Exploratory Meta-Analysis

**ADDITIONAL FILE 2**

**Search Strategy**

Karolin R. Krause^1,2^, Darren B. Courtney^1,3^, Benjamin W. C. Chan^4^, Sarah Bonato^1^, Madison Aitken^1,3^, Jacqueline Relihan^1^, Matthew Prebeg^1^, Karleigh Darnay^1^, Lisa D. Hawke^1,3^, Priya Watson^1,3^, Peter Szatmari^1,3,5^

1. Cundill Centre for Child and Youth Depression, Centre for Addiction and Mental Health (CAMH), Toronto, ON, Canada
2. Evidence-Based Practice Unit, University College London and Anna Freud National Centre for Children and Families and, London, United Kingdom
3. Department of Psychiatry, University of Toronto, Toronto, ON, Canada
4. Department of Family and Community Medicine, University of Toronto, Toronto, ON, Canada
5. Hospital for Sick Children, Toronto, ON, Canada

**Corresponding Author:** Karolin Krause, Cundill Centre for Child and Youth Depression, Centre for Addiction and Mental Health, 80 Workman Way, Toronto, ON M6J 1H4, Canada; Email: Karolin.krause@camh.ca

# Search Strategy

## Search Strategy for Empirical and Theoretical Studies (Search A)

### Medline (OVID)

MEDLINE and Epub Ahead of Print, In-Process & Other Non-Indexed Citations and Daily 1946 to June 23, 2020

| 1 | depressive disorder/ Or depressive disorder, major/ Or dysthymic disorder/ Or (depression or depressive disorder* or dysthymia or depressed).kw,ti. |
| --- | --- |
| 2 | (problem solving or problem-solving).ti, ab, kw. OR exp Problem Solving/ |
| 3 | Psychological Theory/ OR (theor* or concept* or framework* or model*).ti, ab, kw. |
| 4 | exp qualitative research/ or *thematic analysis/ or exp grounded theory/ or interviews as topic/ or focus groups/ or narration/ or (qualitative stud* or “qualitative research” or focus group* or ethnograph* or “key informant” or “interpretative phenomenological analysis” or “narrative analysis” or “thematic analysis” or “grounded theory” or “ideal#type analysis” or “framework analysis”).ti,ab,kw. Or ((“semi?structured” or unstructured or informal or “in?depth” or “face-to-face” or structured or guide) adj3 (interview* or discussion* or questionnaire*)).ti,ab, kw. |
| 5 | (((systematic or state-of-the-art or scoping or literature or umbrella) adj (review* or overview* or assessment*)) or “review* of reviews” or meta-analy* or metaanaly* or ((systematic or evidence) adj1 assess*) or “research evidence” or metasynthe* or meta-synthe*).ti, ab, kw. Or exp Review Literature as Topic/ or exp Review/ or Meta-Analysis as Topic/ or Meta-Analysis/ or “systematic review”/ |
| 6 | “randomized controlled trial”.pt. |
| 7 | (random$ or placebo$ or single blind$ or double blind$ or triple blind$).ti,ab. |
| 8 | (retraction of publication or retracted publication).pt |
| 9 | controlled clinical trial.pt. |
| 10 | Trial.pt. |
| 11 | or/6-10 |
| 12 | (animals not humans).sh. |
| 13 | ((comment or editorial or meta-analysis or practice-guideline or review or letter) not “randomized controlled trial”).pt. |
| 14 | (random sampl$ or random digit$ or random effect$ or random survey or random regression).ti,ab. Not “randomized controlled trial”.pt. |
| 15 | 11 not (12 or 13 or 14) |
| 16 | 1 AND 2 |
| 17 | 3 or 4 or 5 or 15 |
| 18 | 16 AND 17 |
| 19 | limit 18 to (“all child (0 to 18 years)” or “preschool child (2 to 5 years)” or “child (6 to 12 years)” or “adolescent (13 to 18 years)” or “young adult” (19 to 24 years)) |
| 20 | (pediatric* or paediatric* or child* or adolescent? Or youth? Or teenager? Or teen? Or juvenile? Or young adult?).ti,jn. |
| 21 | 18 and 20 |
| 22 | 19 or 21 |
| 23 | limit 22 to english or french or german or spanish |

Similar searches were run in Embase, APA PsycINFO, CINAHL, and Web of Science.

## Expanded Search for Clinical Practice Guidelines (Search B)

### Medline (OVID)

| Database: Ovid MEDLINE: Epub Ahead of Print, In-Process & Other Non-Indexed Citations, Ovid MEDLINE® Daily and Ovid MEDLINE® to June 23, 2020 | |
| --- | --- |
| 1 | exp Depression/ |
| 2 | exp Depressive Disorder/ |
| 3 | depress*.mp. |
| 4 | dysthymic*.mp. |
| 5 | dysthymia*.mp. |
| 6 | exp clinical pathway/ |
| 7 | exp clinical protocol/ |
| 8 | exp consensus/ |
| 9 | exp consensus development conference/ |
| 10 | exp consensus development conferences as topic/ |
| 11 | critical pathways/ |
| 12 | exp guideline/ |
| 13 | guidelines as topic/ |
| 14 | exp practice guideline/ |
| 15 | practice guidelines as topic/ |
| 16 | health planning guidelines/ |
| 17 | treatment guideline*.mp. |
| 18 | (guideline or practice guideline or consensus development conference or consensus development conference, NIH).pt. |
| 19 | (position statement* or policy statement* or practice* parameter* or practise* parameter* or best practice* or best* practise*).mp. |
| 20 | (standard* or guideline*).mp. |
| 21 | ((practice* or practise* or treatment*) adj3 guideline*).mp. |
| 22 | (CPG or CPGs).mp. |
| 23 | Consensus*.mp. |
| 24 | ((critical or clinical or practice* or practise*) adj2 (path or paths or pathway or pathways or protocol*)).mp. |
| 25 | recommendat*.mp. |
| 26 | (care adj2 (standard or path or paths or pathway or pathways or map or maps or plan or plans)).mp. |
| 27 | (algorithm* adj2 (screen* or examination* or test or tested or testing or assess* or diagnosis or diagnoses or diagnosed or diagnosing)).mp. |
| 28 | (algorithm* adj2 (pharmacotherap* or psychopharm* chemotherap* or chemotreatment* or therap* or treat* or interven*)).mp. |
| 29 | exp child/ |
| 30 | exp adolescents/ |
| 31 | exp Young Adult/ |
| 32 | (child* or juvenile* or pubescen* or prepubescen* or pre-pubescen* or teen* or preteen* or tween* or youth* or adoles* or young* adult* or emerg* adult* or pediatric* or paediatric*).mp. |
| 33 | (child* or juvenile* or pubescen* or prepubescen* or pre-pubescen* or teen* or preteen* or tween* or youth* or adoles* or young* adult* or emerg* adult* or pediatric* or paediatric*).jw. |
| 34 | or/1-5 [depression set] |
| 35 | or/6-28 [guideline set] |
| 36 | or/29-33 [child/youth set] |
| 37 | 34 and 35 and 36 |
| 38 | limit 37 to yr="2017 -Current" |
| 39 | limit 38 to (english or french or german or spanish) |
| 40 | limit 37 to yr="2005 -2016" |
| 41 | limit 40 to ( french or german or spanish) |

Similar searches were run in Embase, APA PsycINFO, CINAHL, and Web of Science.

## Grey Literature Search

### Resources published in English

Search terms: varying combinations of the terms “problem-solving” or “problem solving”; “depression”; “adolescents” or “young people” or “children” or “youth”; and same terms for clinical practice guidelines as in original grey literature search.

### Grey literature databases and search engines

BIGG—International database of Grade Guidelines. <https://medlist.paho.org/>. [10/08/2020].

Clinical Practice Guidelines Portal (Australia). <https://www.clinicalguidelines.gov.au/portal>. [10/08/2020].

ECRI Guidelines Trust. <https://guidelines.ecri.org/>. [10/08/2020].

Evidence Aid. <https://evidenceaid.org/resource/>. [10/08/2020].

G-I-N Guidelines International Network. <https://g-i-n.net/library/international-guidelines-library>. [10/08/2020].

Grey Literature Report. [www.greylit.org](http://www.greylit.org). [26/06/2020].

Joule CPG Infobase. [https ://joulecma.ca/cpg/homepage](https://joulecma.ca/cpg/homepage). [10/08/2020].

MAGIC. [https ://app.magicapp.org/](https://app.magicapp.org/). [10/08/2020].

MedNar search engine for government and organisational websites. <https://mednar.com/mednar/desktop/en/search.html>. [26/06/2020].

Open-Grey. [www.opengrey.eu](http://www.opengrey.eu). [26/06/2020].

The Royal College of Psychiatrists Clinical Guideline Directory (UK). <https://www.rcpch.ac.uk/resources/clinical-guideline-directory>. [10/08/2020].

Trip database. <https://www.tripdatabase.com/>. [10/08/2020].

### Governmental or organizational websites

Anna Freud National Centre for Children and Families (UK). <https://www.annafreud.org/on-my-mind/self-care/problem-solving/>. [09/07/2020].

American Academy of Child and Adolescent Psychiatry (USA). <http://www.aacap.org/>. [09/07/2020].

Association of Psychiatrists in Nigeria (Nigeria). <https://www.apn.org.ng/>. [09/07/2020].

Basic Needs. <http://www.basicneeds.org/>. [09/07/2020].

British Psychological Society (UK). <https://www.bps.org.uk/>. [09/07/2020].

Canadian Academy of Child and Adolescent Psychiatry (Canada). <https://www.cacap-acpea.org/?s=problem-solving+depression>. [09/07/2020].

Canadian Paediatric Society (Canada). <http://www.cps.ca/en/>. [09/07/2020].

Canadian Psychological Association (Canada). <https://cpa.ca/?s=problem-solving+depression>. [09/07/2020].

Centre for Addiction and Mental Health (Canada). <https://www.camh.ca/en/>. [09/07/2020].

Department of Health (South Africa). <http://www.health.gov.za/>. [09/07/2020].

Federal Ministry of Health (Nigeria). <https://www.health.gov.ng/>. [09/07/2020].

Jack.org (Canada). <https://jack.org/Search-Result?searchtext=problem-solving&searchmode=anyword> [26/06/2020].

Jigsaw (Ireland). <https://www.jigsaw.ie/>. [09/07/2020].

Indian Psychiatric Society (India). <https://indianpsychiatricsociety.org/ips-guidelines/>. [09/07/2020].

Headspace (Australia). <https://headspace.org.au/>. [09/07/2020].

Mental Health Authority Ghana (Ghana). <https://mhaghana.com/about-us>/. [09/07/2020].

Mental Health Foundation (UK). <https://www.mentalhealth.org.uk/search/?query=problem-solving&op=Search> [09/07/2020].

Mental Health Nigeria (Nigeria). <http://mentalhealthnigeria.org/>. [09/07/2020].

Ministry of Health (Gambia). <http://www.moh.gov.gm/>. [09/07/2020].

Ministry of Health (Ghana). <https://www.moh.gov.gh/>. [09/07/2020].

Ministry of Health and Population (Malawi). <https://www.health.gov.mw/>. [09/07/2020].

Ministry of Health (Kenya). <https://www.health.go.ke/>. [09/07/2020].

Ministry of Health (Rwanda). <https://moh.gov.rw/>. [09/07/2020].

Ministry of Health (Uganda). <https://www.health.go.ug/>. [09/07/2020].

Ministry of Health (Zambia). <https://www.moh.gov.zm/>. [09/07/2020].

Ministry of Health and Child Care (Zimbabwe). <http://www.mohcc.gov.zw/>. [09/07/2020].

Ministry of Health, Community Development, Gender, Elderly and Children (Tanzania). <https://www.moh.go.tz/en/>. [09/07/2020].

Ministry of Health and Family Welfare (India). <https://www.mohfw.gov.in/>. [09/07/2020].

Ministry of Health and Sanitation (Sierra Leone). <https://mohs.gov.sl/>. [09/07/2020].

Ministry of Health and Wellness (Botswana). <https://www.moh.gov.bw/> [09/07/2020, security certificate expired].

Sangath (India). <https://www.sangath.in/>. [09/07/2020].

Substance Abuse and Mental Health Services Administration (SAMHSA, USA). <https://store.samhsa.gov/>. [09/07/2020].

South African Society of Psychiatrists (South Africa). <https://www.sasop.co.za/resources-publications>. [09/07/2020].

The Kenya Psychiatric Association (Kenya). <https://www.kenyapsychiatrist.org/>. [09/07/2020].

The Royal College of Psychiatrists (UK). [https://www.rcpch.ac.uk/resources/](https://www.rcpch.ac.uk/resources/clinical-guideline-directory) . [09/07/2020].

The Royal Australian and New Zealand College of Psychiatrists (Australia & New Zealand). <https://www.ranzcp.org/news-policy/policy-and-advocacy/reports> [09/07/2020].

Uganda Psychiatric Association (Uganda). [https ://www.ugandapsychiatricassociation.com/#](https://www.ugandapsychiatricassociation.com/). [09/07/2020].

World Health Organization. (<https://www.who.int/>). [09/07/2020].

Young Minds (UK). <https://youngminds.org.uk/search-results/?terms=problem+solving&category=Publications> [09/07/2020].

## Resources published in French

Search terms : thérapie de résolution de problèmes, « résolution de problèmes », « depression », « adolescents », « jeunes », « directrices pour/sur la pratique clinique », « directives de pratique clinique », « guides de pratique clinique »

Association des Psychiatres de secteur Infanto-juvénile (France). [http ://www.api.asso.fr/](http://www.api.asso.fr/), [09/07/2020].

Centre de Référence en Santé Mentale (Belgium). [https ://www.cresam.be/](https://www.cresam.be/). [09/07/2020].

Centre Fédéral d’Expertise des Soins de Santé (Belgium). [https ://kce.fgov.be/fr](https://kce.fgov.be/fr). [09/07/2020].

Centre National Hospitalier Universitaire de Cotonou (Benin). [http ://www.cnhu-hkm.org/](http://www.cnhu-hkm.org/). [09/07/2020].

Haute Autorité de Santé (France). <https://www.has-sante.fr/jcms/fc_2875171/en/resultat-de-recherche-antidot-2019?text=depression&tmpParam=typesf%3Dguidelines&typesf=guidelines&opSearch=&lang=en> [09/07/2020].

La SSMG (Belgium). [https ://www.ssmg.be/guides-de-pratique-clinique/](https://www.ssmg.be/guides-de-pratique-clinique/). [09/07/2020].

Ministère de la Sante (Benin). [https ://sante.gouv.bj/](https://sante.gouv.bj/). [09/07/2020].

Ministère de la Sante et de l’Action Sociale (Sénégal). [http ://www.sante.gouv.sn/](http://www.sante.gouv.sn/). [09/07/2020, site down].

Ministère de la Sante et des Affaires Sociales (Mali). [http ://www.sante.gov.ml/](http://www.sante.gov.ml/). [09/07/2020].

Ministère de la Sante, de la population, de la promotion de la femme et de l’intégration de la femme au développement (République du Congo). [http ://sante.gouv.cg/](http://sante.gouv.cg/). [09/07/2020].

Ministère de la Santé Publique (Cameroon). [https ://www.minsante.cm/](https://www.minsante.cm/) [09/07/2020].

Service public fédéral Sante Publique, Sécurité de la Chaine Alimentaire et Environnement (Belgium). [https ://www.health.belgium.be/fr](https://www.health.belgium.be/fr). [09/07/2020].

Société Française de Psychiatrie de l’Enfant et de l’Adolescent et disciplines associes, [http ://sfpeada.fr/](http://sfpeada.fr/) [09/07/2020].

## Resources published in German

Search terms: varying combinations of the terms „Problemlösungstherapie“, „Problemlösung“, „Leitlinie“; „Jugendpsychiatrie“, „Jugendpsychologie“, „Depression“, „Jugendliche“

Bundesministerium fuer Gesundheit (Germany). <https://www.bundesgesundheitsministerium.de/>. [09/07/2020].

Bundesamt fuer Gesundheit (Swizterland). <https://www.bag.admin.ch/bag/de/home.html>. [09/07/2020].

Bundestministerium Soziales, Gesundheit, Pflege und Konsumentenschutz (Austria). <https://www.sozialministerium.at/Themen/Gesundheit.html>. [09/07/2020].

German Association for Psychiatry, Psychotherapy and Psychosomatics (Germany). <https://www.dgppn.de/en/> [09/07/2020].

Stiftung Deutsche Depressionshilfe (Germany). <https://www.deutsche-depressionshilfe.de/suche?keywords=Probleml%C3%B6sung>. [09/07/2020].

Deutsche Gesellschaft für Kinder- und Jugendpsychatrie, Psychosomatik und Psychotherapie e.V. (Germany). <https://www.dgkjp.de/> [09/07/2020; website offline]

Berufsverband für Kinder- und Jugendpsychiatrie, Psychosomatik und Psychotherapie in Deutschland e. V. & Bundesarbeitsgemeinschaft der Leitenden Klinikärzte für Kinder- und Jugendpsychiatrie, Psychosomatik und Psychotherapie e. V. (Germany). <https://www.kinderpsychiater.org/startseite/> [09/07/2020].

Der Österreichische Bundesverband für Psychotherapie (Austria) <https://www.psychotherapie.at/oebvp/arbeitsbereiche/saeuglings-kinder-und-jugendpsychotherapie> [09/07/2020].

Arbeitsgemeinschaft der Wissenschaftlichen Medizinischen Fachgesellschaften e.V. (Germany). <https://www.awmf.org/leitlinien/leitlinien-suche.html> [09/07/2020].

Schweizerische Gesellschaft für Kinder- und Jugendpsychiatrie und -psychotherapie (Swizterland), <https://www.sgkjpp.ch/> [09/07/2020].

## Resources Published in Spanish

Search terms: varying combinations of the terms “terapia de resolución de problemas”, “resolución de problemas”, “adolescentes”, “juvenil”, “depresión”, “guía clínica”

Asociación Española de Pediatría (AEP) <https://www.aeped.es/> [09/07/2020].

Asociación Española de Psiquiatría del Niño y el Adolescente; <https://aepnya.es/> [09/07/2020].

Asociación de Psiquiatras Argentinos (APSA). <https://www.apsa.org.ar/>. [09/07/2020].

Asociación Psiquiátrica Peruana (Peru). <https://www.app.org.pe/>. [09/07/2020].

Asociación Mexicana de Psiquiatría Infantil (AMPI AC) <http://www.ampi-ac.org/index.html> [09/07/2020].

Departmento de Salud (Puerto Rico). <http://www.salud.gov.pr/Pages/Home.aspx>. [09/07/2020].

GuíaSalud, <https://portal.guiasalud.es/gpc/depresion-infancia/> [09/07/2020].

<https://www.gob.mx/cms/uploads/attachment/file/452952/3._Depresi_n_en_Ni_os_y_Adolescentes..pdf> [09/07/2020].

Ministerio del Poder Popular para la Salud (Venezuela). <http://www.mpps.gob.ve/>. [09/07/2020].

Ministero de Salud (Argentina). <https://www.argentina.gob.ar/salud>. [09/07/2020].

Ministerio de Salud (Colombia). <https://www.minsalud.gov.co/> [09/07/2020].

Ministero de Salud (Chile). <https://www.minsal.cl/portal/url/item/7222754637c08646e04001011f014e64.pdf> [09/07/2020].

Ministerio de Salud (Bolivia). <https://www.minsalud.gob.bo/> [09/07/2020].

Ministerio de Salud (Ecuador). <https://www.salud.gob.ec/>. [09/07/2020].

Ministerio de Salud (Peru). <https://www.gob.pe/minsa/>. [09/07/2020].

Ministerio de Salud de El Salvador (El Salvador). <https://www.salud.gob.sv/>. [09/07/2020].

Ministerio de Salud de Nicaragua (Nicaragua). [www.minsa.ni](http://www.minsa.ni). [09/07/2020; site could not be reached].

Ministerio de Salud de Panama (Panama). <http://www.minsa.gob.pa/>. [09/07/2020].

Ministerio de Salud de Costa Rica (Costa Rica). <https://www.ministeriodesalud.go.cr/>. [09/07/2020].

Ministerio de Salud Pública (Uruguay). <https://www.gub.uy/ministerio-salud-publica/>. [09/07/2020].

Ministerio de Salud Pública y Asistencia Social (Guatemala). <https://www.mspas.gob.gt/>. [09/07/2020].

Ministerio de Salud Pública y Asistencia Social (Dominican Republic). <https://www.msp.gob.do/web/>. [09/07/2020].

Ministerio de Salud Pública y Bienestar Social (Paraguay). <https://www.mspbs.gov.py/index.php>. [09/07/2020].

Salud Quilota (Chile). <https://www.saludquillota.cl/web/index.html>. [09/07/2020].

Secretaría de Salud Honduras (Honduras). <http://www.salud.gob.hn/site/>. [09/07/2020].

Sociedad Española de Psiquiatría y Psicoterapia del Niño y del Adolescente, <https://www.sepypna.com/> [09/07/2020].

Sociedad de Psiquiatria del Uruguay (Uruguay). <http://spu.org.uy/>. [09/07/2020].
