# Supplementary material for: Problem-solving training as an active ingredient of treatment for youth depression: a scoping review and exploratory meta-analysis
Source: BMC Psychiatry. 2021 Aug 24;21:397. doi: 10.1186/s12888-021-03260-9 (PMC8383463; doi:10.1186/s12888-021-03260-9)
Supplement: Supplementary file 3 — Additional file 3. List of Studies Included in the Scoping Review. [file 12888_2021_3260_MOESM3_ESM.docx]

#

Problem-Solving Training as an Active Ingredient of Treatment for Youth Depression: A Scoping Review and Exploratory Meta-Analysis

**ADDITIONAL FILE 3**

**List of Studies Included in the Scoping Review**

Karolin R. Krause^1,2^, Darren B. Courtney^1,3^, Benjamin W. C. Chan^4^, Sarah Bonato^1^, Madison Aitken^1,3^, Jacqueline Relihan^1^, Matthew Prebeg^1^, Karleigh Darnay^1^, Lisa D. Hawke^1,3^, Priya Watson^1,3^, Peter Szatmari^1,3,5^

1. Cundill Centre for Child and Youth Depression, Centre for Addiction and Mental Health (CAMH), Toronto, ON, Canada
2. Evidence-Based Practice Unit, University College London and Anna Freud National Centre for Children and Families and, London, United Kingdom
3. Department of Psychiatry, University of Toronto, Toronto, ON, Canada
4. Department of Family and Community Medicine, University of Toronto, Toronto, ON, Canada
5. Hospital for Sick Children, Toronto, ON, Canada

**Corresponding Author:** Karolin Krause, Cundill Centre for Child and Youth Depression, Centre for Addiction and Mental Health, 80 Workman Way, Toronto, ON M6J 1H4, Canada; Email: Karolin.krause@camh.ca

# List of Studies Included in the Scoping Review

## Randomized Control Trials of Problem-Solving Therapy

Eskin, M., Ertekin, K., & Demir, H. (2008). Efficacy of a problem-solving therapy for depression and suicide potential in adolescents and young adults. *Cognitive Therapy and Research*, *32*(2), 227–245. <https://doi.org/10.1007/s10608-007-9172-8>

Hoek, W., Schuurmans, J., Koot, H. M., & Cuijpers, P. (2012). Effects of Internet-Based Guided Self-Help Problem-Solving Therapy for Adolescents with Depression and Anxiety: A Randomized Controlled Trial. *PLoS ONE*, *7*(8), 1–7. <https://doi.org/10.1371/journal.pone.0043485>

Michelson, D., Malik, K., Parikh, R., Weiss, H. A., Doyle, A. M., Bhat, B., Sahu, R., Chilhate, B., & Mathur, S. (2020). Effectiveness of a brief lay counsellor-delivered , problem- solving intervention for adolescent mental health problems in urban , low-income schools in India : a randomised controlled trial. *The Lancet Child and Adolescent Health*, *4642*(20). <https://doi.org/10.1016/S2352-4642(20)30173-5>

Parker, A. G., Hetrick, S. E., Jorm, A. F., Mackinnon, A. J., McGorry, P. D., Yung, A. R., Scanlan, F., Stephens, J., Baird, S., Moller, B., & Purcell, R. (2016). The effectiveness of simple psychological and physical activity interventions for high prevalence mental health problems in young people: A factorial randomised controlled trial. *Journal of Affective Disorders*, *196*, 200–209. <https://doi.org/10.1016/j.jad.2016.02.043>

## Secondary Analyses Examining Problem-Solving-Related Concepts as Predictors, Moderators, or Mediators of Treatment Response

Becker-Weidman, E. G., Jacobs, R. H., Reinecke, M. A., Silva, S. G., & March, J. S. (2010). Social problem-solving among adolescents treated for depression. *Behaviour Research and Therapy*, *48*(1), 11–18. https://doi.org/10.1016/j.brat.2009.08.006

Dietz, L. J., Marshal, M. P., Burton, C. M., Bridge, J. A., Birmaher, B., Kolko, D., Duffy, J. N., & Brent, D. A. (2014). Social problem solving among depressed adolescents is enhanced by structured psychotherapies. *Journal of Consulting and Clinical Psychology*, *82*(2), 202–211. <https://doi.org/10.1037/a0035718>

Kaufman, N. K., Rohde, P., Seeley, J. R., Clarke, G. N., & Stice, E. (2005). Potential mediators of cognitive-behavioral therapy for adolescents with comorbid major depression and conduct disorder. *Journal of Consulting and Clinical Psychology*, *73*(1), 38–46. <https://doi.org/10.1037/0022-006X.73.1.38>

Kaufman, N. K., Rohde, P., Seeley, J. R., Clarke, G. N., & Stice, E. (2005). Potential mediators of cognitive-behavioral therapy for adolescents with comorbid major depression and conduct disorder. *Journal of Consulting and Clinical Psychology*, *73*(1), 38–46. <https://doi.org/10.1037/0022-006X.73.1.38>

## Clinical Practice Guidelines

Birmaher, B., & Brent, D. (2007). Practice parameter for the assessment and treatment of children and adolescents with depressive disorders. *Journal of the American Academy of Child and Adolescent Psychiatry*, *46*(11), 1503–1526. https://doi.org/10.1097/chi.0b013e318145ae1c

Bostic, J. Q., Rubin, D. H., Prince, J., & Schlozman, S. (2005). Treatment of Depression in Children and Adolescents. *Journal of Psychiatric Practice*, *11*(3), 141–154.

Cheung, A. H., Zuckerbrot, R. A., Jensen, P. S., Laraque, D., & Stein, R. E. K. (2018). Guidelines for adolescent depression in primary care (GLAD-PC): Part II. Treatment and ongoing management. *Pediatrics*, *141*(3), e20174082. https://doi.org/10.1542/peds.2017-4082

Cincinnati Children’s Hospital Medical Centre. (2010). *Best Evidence Statement (BESt): Treatment of children and adolescents with Major Depressive Disorder (MDD) during the Acute Phase*. https://doi.org/10.1002/pits.20620.

Dolle, K., & Schulte-Körne, G. (2013). The treatment of depressive disorders in children and adolescents. *Deutsches Arzteblatt International*, *110*(50). https://doi.org/10.3238/arztebl.2013.0854

Driot, D., Nguyen-Soenen, J., Costes, M., Pomier, M., Birebent, J., Oustric, S., & Dupouy, J. (2020). Management of child and adolescent depression in primary care: A systematic meta-review. *Encephale*, *46*(1), 41–54. https://doi.org/10.1016/j.encep.2019.07.014

Falcato, M. A., Muñoz, N. H., & Urquiola, Y. C. (2011). Depresión en la adolescencia: consideraciones necesarias para su diagnóstico y tratamiento. In *Revista Finlay* (Vol. 7, Issue 3). http://scielo.sld.cu/scielo.php?script=sci_arttext&pid=S2221-24342017000300001

Gallagher, R. (2005). Evidence ­ Based Psychotherapies for Depressed Adolescents: A Review and Clinical Guidelines. *Primary Psychiatry*, *12*(9), 33.

Grover, S., & Avasthi, A. (2019). Clinical practice guidelines for the management of depression in children and adolescents. *Indian Journal of Psychiatry*, *61*(8), S226–S240. https://doi.org/10.4103/psychiatry.IndianJPsychiatry_563_18

Grupo de trabajo de la actualización de la Guía de Práctica Clínica sobre la Depresión Mayor en la Infancia y la Adolescencia. (2018). Guía de Práctica Clínica sobre la Depresión Mayor en la Infancia y en la Adolescencia. Actualización. In *Guías de Práctica Clínica en el SNS*. https://portal.guiasalud.es/wp-content/uploads/2018/12/GPC_575_Depresion_infancia_Avaliat_compl.pdf

Guidelines and Protocols Advisory Committee. (2010). Anxiety and Depression in Children and Youth - Diagnosis and Treatment. In *British Columbia Guidelines & Protocols*. https://www2.gov.bc.ca/assets/gov/health/practitioner-pro/bc-guidelines/depressyouth.pdf

Haute Autorité de Santé. (2014). *Recommandation de bonne pratique - Manifestations dépressives à l’adolescence: repérage, diagnostic et prise en charge en soins de premier recours*. https://www.has-sante.fr/portail/upload/docs/application/pdf/2014-12/manifestations_depressives_recommandations.pdf

Kaiser Permanente. (2018). Adult and Adolescent Depression Screening, Diagnosis, and Treatment Guideline. In *Kaiser Foundation Health Plan of Washington* (Issue January). https://wa.kaiserpermanente.org/static/pdf/public/guidelines/depression.pdf

MacQueen, G. M., Frey, B. N., Ismail, Z., Jaworska, N., Steiner, M., Lieshout, R. J. V., Kennedy, S. H., Lam, R. W., Milev, R. V., Parikh, S. V., & Ravindran, A. V. (2016). Canadian Network for Mood and Anxiety Treatments (CANMAT) 2016 clinical guidelines for the management of adults with major depressive disorder: Section 6. Special populations: Youth, women, and the elderly. *Canadian Journal of Psychiatry*, *61*(9), 588–603. https://doi.org/10.1177/0706743716659276

Ministerio de Salud. (2013). *Guía Clínica para el tratamiento de adolescentes de 10 a 14 años con Depresión.* https://www.minsal.cl/portal/url/item/e11791fc480273e9e040010164014e60.pdf

Ministerio de Salud. (2013). *Guía Clínica Depresión en personas de 15 años y más*. https://www.minsal.cl/portal/url/item/7222754637c08646e04001011f014e64.pdf

Nogales Imaca, A. I., Rodríguez Juárez, H., Cortés Meda, G. M., Cabrera Abud, I. I., Esperón Vargas, C., Lohman Alamilla, K., & Peñaloza Torres, E. C. (n.d.). Guía Clínica: Depresión en niños y adolescentes. In *Guías Clínicas del Hospital Psiquiátrico Infantil “Dr. Juan N. Navarro.”*

Orygen. (2017). Treating depression in young people: Guidance, resources and tools for assessment and management. In *Orygen*. https://www.orygen.org.au/Training/Resources/Depression/Clinical-practice-points/Treating-depression-in-yp

Seok Seo, J., Rim Song, H., Bin Lee, H., Park, Y. M., Hong, J. W., Kim, W., Wang, H. R., Lim, E. S., Jeong, J. H., Jon, D. I., Joon Min, K., Sup Woo, Y., & Bahk, W. M. (2014). The Korean medication algorithm for depressive disorder: Second revision. *Journal of Affective Disorders*, *167*, 312–321. https://doi.org/10.1016/j.jad.2014.05.031

The National Institute for Health and Care Excellence [NICE]. (2019). Depression in children and young people: Identification and management. In *NICE guideline*. https://doi.org/10.1211/CP.2018.20204575

US Preventive Services Task Force. (2009). Screening and treatment for major depressive disorder in children and adolescents: US Preventive Services Task Force recommendation statement. *Pediatrics*, *123*(4), 1223–1228. https://doi.org/10.1542/peds.2008-2381

World Health Organization. (2015). *Update of the Mental Health Gap Action Programme (mhGAP) guidelines for mental, neurological and substance use disorders* (Issue May). https://www.who.int/maternal_child_adolescent/documents/health-promotion-interventions/en/

Zuckerbrot, R. A., Cheung, A., Jensen, P. S., Stein, R. E. K., & Laraque, D. (2018). Guidelines for adolescent depression in primary care (GLAD-PC): Part I. Practice preparation, identification, assessment, and initial management. *Pediatrics*, *141*(3). <https://doi.org/10.1542/peds.2017-4081>

## Conceptual Studies (considered in the introductory background section)

Chang, E. C., D’Zurilla, T. J., & Sanna, L. J. (2004). *Social problem solving: Theory, research, and training.* American Psychological Association.

Diamond, G., & Siqueland, L. (1995). Family Therapy for the treatment of depressed adolescents. *Psychotherapy*, *32*(1), 77–90. <https://doi.org/10.1037/0033-3204.32.1.77>

D’Zurilla, T. J., Chang, E. C., & Sanna, L. J. (2004). Social Problem Solving: Current Status and Future Directions. *Social Problem Solving: Theory, Research, and Training.*, 241–253. <https://doi.org/10.1037/10805-014>

D’Zurilla, T. J., & Goldfried, M. R. (1971). Problem solving and behavior modification. *Journal of Abnormal Psychology*, *78*(1), 107–126. <https://doi.org/10.1037/h0031360>

D’Zurilla, T. J., Nezu, A. M., & Maydeu-Olivares, A. (2004). Social Problem Solving: Theory and Assessment. In E. C. Chang, T. J. D’Zurilla, & L. J. Sanna (Eds.), *Social problem solving: Theory, research, and training.* (pp. 11–27). American Psychological Association. https://doi.org/10.1037/10805-001

D’Zurilla, T. J., & Nezu, A. M. (2007). *Problem-solving therapy: a positive approach to clinical intervention* (3rd ed.). Spring Publishing Company.

D’Zurilla, T. J., & Nezu, A. M. (2010). Problem-solving therapy. In *Handbook of Cognitive-Behavioral Therapies* (Third, pp. 197–225). Guilford Press.

Nezu, A. M. (1987). A problem-solving formulation of depression: A literature review and proposal of a pluralistic model. *Clinical Psychology Review*, *7*(2), 121–144. https://doi.org/10.1016/0272-7358(87)90030-4

Nezu, A. M. (2004). Problem solving and behavior therapy revisited. *Behavior Therapy*, *35*(1), 1–33. <https://doi.org/10.1016/S0005-7894(04)80002-9>

Nezu, A. M., Nezu, C. M., & D’Zurilla, T. J. (2013). Problem-solving therapy: A treatment manual. In *Problem-solving therapy: A treatment manual.* Springer Publishing Company.

Nezu, A. M., & Perri, M. G. (1989). Social Problem-Solving Therapy for Unipolar Depression: An Initial Dismantling Investigation. *Journal of Consulting and Clinical Psychology*, *57*(3), 408–413. https://doi.org/10.1037/0022-006X.57.3.408

Ugueto, A. M., Santucci, L. C., Krumholz, L. S., & Weisz, J. R. (2014). Problem-Solving Skills Training. In E. S. Sburlati, H. J. Lyneham, C. A. Schniering, & R. M. Rapee (Eds.), *Evidence-Based CBT for Anxiety and Depression in Children and Adolescents: A Competencies-Based Approach* (pp. 247–259). John Wiley & Sons. <https://doi.org/10.1002/9781118500576>
