# Supplementary material for: Problem-solving training as an active ingredient of treatment for youth depression: a scoping review and exploratory meta-analysis
Source: BMC Psychiatry. 2021 Aug 24;21:397. doi: 10.1186/s12888-021-03260-9 (PMC8383463; doi:10.1186/s12888-021-03260-9)
Supplement: Supplementary file 4 — Additional file 4. Characteristics of Included Clinical Practice Guidelines. [file 12888_2021_3260_MOESM4_ESM.docx]

Problem-Solving Training as an Active Ingredient of Treatment for Youth Depression: A Scoping Review and Exploratory Meta-Analysis

**ADDITIONAL FILE 4**

**Characteristics of Included Clinical Practice Guidelines**

Karolin R. Krause^1,2^, Darren B. Courtney^1,3^, Benjamin W. C. Chan^4^, Sarah Bonato^1^, Madison Aitken^1,3^, Jacqueline Relihan^1^, Matthew Prebeg^1^, Karleigh Darnay^1^, Lisa D. Hawke^1,3^, Priya Watson^1,3^, Peter Szatmari^1,3,5^

1. Cundill Centre for Child and Youth Depression, Centre for Addiction and Mental Health (CAMH), Toronto, ON, Canada
2. Evidence-Based Practice Unit, University College London and Anna Freud National Centre for Children and Families and, London, United Kingdom
3. Department of Psychiatry, University of Toronto, Toronto, ON, Canada
4. Department of Family and Community Medicine, University of Toronto, Toronto, ON, Canada
5. Hospital for Sick Children, Toronto, ON, Canada

**Corresponding Author:** Karolin Krause, Cundill Centre for Child and Youth Depression, Centre for Addiction and Mental Health, 80 Workman Way, Toronto, ON M6J 1H4, Canada; Email: Karolin.krause@camh.ca

**Characteristics of Included Clinical Practice Guidelines (CPGs)**

**Table S1.** **Characteristics of Included Clinical Practice Guidelines and Coverage of Problem-Solving**

| **Reference** | **Year** | **Country** | **Issuing Organisation** | **Age Group (s)** | **Target Disorder (s)** | **Mention of Problem-Solving (with page number in the primary document)** |
| --- | --- | --- | --- | --- | --- | --- |
| Nogales Imaca et al. (1) | No date | Mexico | Government | Children and adolescents | Depression | PS training described as a component of CBT that contributes to its effectiveness in children and adolescents with moderate or severe depression and / or with psychiatric comorbidities (p. 19-20). |
| Driot et al. (2) | 2020 | France | N/A | 6-18 yrs | Depression | No coverage |
| Grover & Avasthi (3) | 2019 | India | Speciality society | Up to 24 yrs | Depression | Problem-solving described as a basic component of psychoeducation (p. S235). |
| NICE (4) | 2019 | United Kingdom | Government | 5-18 yrs | Depression | 2019: Problem-solving described as a common component of digital CBT (pg. 45) |
| Cheung et al. (5) | 2018 | United States | Independent expert group & speciality society | 10-21 yrs | Depression | Problem-solving skills described as an essential component of CBT “to reduce feelings of hopelessness”; improving interpersonal problem-solving skills is described as an essential component of IPT (2018, pg. 8). |
| Kaiser Permanente (6) | 2018 | United States | Health care provider | 12 yrs and older | Depression | Avoidance of problem solving described as one type of behaviour that psychotherapy typically aims to change. (p. 9) |
| Grupo de trabajo (7) | 2018 | Spain | Government | 5-18 yrs | Depression | Problem-solving described as a “classic technique” that forms a component of Dialectical Behaviour Therapy (DBT; pg. 113) |
| Orygen (8) | 2017 | Australia | Health care provider | 12-25 yrs | Depression | For persistent sub-threshold depressive symptoms, or mild to moderate depression, the guidelines recommends “6-8 sessions of individual guided self-help based on the principles of CBT including behavioural activation and problem-solving techniques over 9-12 weeks” (p. 16). |
| Falcato et al. (9) | 2017 | Cuba | Health care provider | Adolescents (nfs) | Depression | No coverage |
| MacQueen et al. (10) | 2016 | Canada | Independent expert group | Youth, Women, and the Elderly | Depression | No coverage in relation to youth |
| World Health Organization (11) | 2015 | International | International Organization | All ages | Mental health disorders | PS training is recommended as an adjunct treatment (for example in combination with ant depressive medication) for the treatment of depression in older adolescents and adults (p. 27). |
| Haute Autorité de Santé (12) | 2014 | France | Other | 12-18 yrs | Depression | Problem-solving (i.e., clarifying problems and helping to solve them) is described as a component of supportive therapy (pg. 22). |
| Dolle & Schulte-Körne (13,14) | 2013 | Germany | Specialty society | 3-18 yrs | Depression | No coverage |

| MINSAL (15) | 2013 | Chile | Government | 15 yrs and older | Depression | Problem-solving skills are described as a component of CBT (pg. 93).  For mild depression, it is suggested that the most effective treatment is supportive clinical care, complemented by psychoeducation and problem solving-tools or supportive counselling (pg. 126).  Suggests that the determinants of the extent to which problem-solving therapy is effective are un-known (in adults; pg. 60). |
| --- | --- | --- | --- | --- | --- | --- |
| MINSAL (16) | 2013 | Chile | Government | 10-14 yrs | Depression | No coverage |
| Ministry of Health (17) | 2012 | Singapore | Government | All ages | Depression | No coverage in relation to youth. |
| BeyondBlue (18) | 2011 | Australia | Other | 13-24 yrs | Depression | Problem-solving only discussed in the context of prevention.  “Skills and knowledge taught in most CBT interventions include psychoeducation, self-monitoring and evaluation, social skills, participating in pleasant activities, relaxation, constructive thinking, self-reinforcement, communication, negotiation and problem solving” (p. 52).  “Strategies employed in IPT include education; clarification of feelings, expectations and roles; and facilitation of social competence. Techniques include communication analysis, interpersonal problems solving, modelling and role-playing.” (p. 52) |
| Cincinnati Children’s Hospital Medical Centre (19) | 2010 | United States | Health care provider | 6-17 yrs | Depression | Supportive therapy is recommended as the initial treatment for adolescents with “brief, uncomplicated and/or mild depression continuing for 4 to 8 sessions until remission”, with problem-solving described as an element of supportive psychotherapy (pg. 1) |
| Guidelines and Protocols Advisory Committee (20) | 2010 | Canada | Government & speciality society | Up to 18 yrs | Anxiety & Depression | Suggests that problem-solving is part of healthy thinking patterns, which can help reduce introspection, self-criticism, rumination, or dysfunctional thoughts that can exacerbate depression (Appendix B).  Interpersonal problem solving is described as a component of IPT (p. 4). |
| US Preventive Services Task Force (USPSTF) (21) | 2009 | United States | Independent expert group | 7-18 yrs | Depression | No coverage |
| Birmaher & Brent (22) | 2007 | United States | Speciality society | Children and adolescents (nfs) | Depression | Suggests that each phase of treatment should involve supportive management: “In addition to psychoeducation, all subjects require supportive psychotherapeutic management, which may include active listening and reflection, restoration of hope, problem solving, coping skills, and strategies for maintaining participation in treatment” (p.1510). |
| Gallagher (23) | 2005 | United States | Other | Adolescents (nfs) | Depression | PS training described as a component of CBT, Systemic-Behavioural Family Therapy, and IPT.  “Instruction and practice in problem solving skills helps the adolescent alter outcomes and can provide hope. The depressed adolescent can be taught to approach difficult situations more effectively by learning to generate alternative ideas, evaluate their potential consequences, and implement solutions that seem most desirable.” (no page numbers provided). |
| Bostic et al. (24) | 2005 | United States | N/A | Children and adolescents (nfs) | Depression | No coverage |

*Note.* NFS = not further specified.

**References**

1. Nogales Imaca AI, Rodríguez Juárez H, Cortés Meda GM, Cabrera Abud II, Esperón Vargas C, Lohman Alamilla K, et al. Guía Clínica: Depresión en niños y adolescentes. Guías Clínicas del Hospital Psiquiátrico Infantil “Dr. Juan N. Navarro.”

2. Driot D, Nguyen-Soenen J, Costes M, Pomier M, Birebent J, Oustric S, et al. Prise en charge de la dépression de l’enfant et l’adolescent en soins premiers : une métarevue systématique de la littérature. Encephale. 2020 Feb;46(1):41–54.

3. Grover S, Avasthi A. Clinical practice guidelines for the management of depression in children and adolescents. Indian J Psychiatry. 2019;61(8):226.

4. The National Institute for Health and Care Excellence [NICE]. Depression in children and young people: identification and management (NICE Guideline No. 134) [Internet]. 2019. Available from: https://www.nice.org.uk/guidance/ng134/chapter/Recommendations#steps-4-and-5-managing-moderate-to-severe-depression

5. Cheung AH, Zuckerbrot RA, Jensen PS, Laraque D, Stein REK. Guidelines for Adolescent Depression in Primary Care (GLAD-PC): Part II. Treatment and Ongoing Management. Pediatrics. 2018;141(3):e20174082.

6. Kaiser Permanente. Adult and Adolescent Depression Screening, Diagnosis, and Treatment Guideline [Internet]. Kaiser Foundation Health Plan of Washington. 2018. Available from: https://wa.kaiserpermanente.org/static/pdf/public/guidelines/depression.pdf

7. Grupo de trabajo de la actualización de la Guía de Práctica Clínica sobre la Depresión Mayor en la Infancia y la Adolescencia. Guía de Práctica Clínica sobre la Depresión Mayor en la Infancia y en la Adolescencia. Actualización [Internet]. Guías de Práctica Clínica en el SNS. 2018. Available from: https://portal.guiasalud.es/wp-content/uploads/2018/12/GPC_575_Depresion_infancia_Avaliat_compl.pdf

8. Orygen. Treating depression in young people: Guidance, resources and tools for assessment and management [Internet]. Orygen. 2017. Available from: https://www.orygen.org.au/Training/Resources/Depression/Clinical-practice-points/Treating-depression-in-yp

9. Falcato MA, Muñoz NH, Urquiola YC. Depresión en la adolescencia: consideraciones necesarias para su diagnóstico y tratamiento [Internet]. Vol. 7, Revista Finlay. 2011. Available from: http://scielo.sld.cu/scielo.php?script=sci_arttext&pid=S2221-24342017000300001

10. MacQueen GM, Frey BN, Ismail Z, Jaworska N, Steiner M, Lieshout RJV, et al. Canadian Network for Mood and Anxiety Treatments (CANMAT) 2016 clinical guidelines for the management of adults with major depressive disorder: Section 6. Special populations: Youth, women, and the elderly. Can J Psychiatry. 2016;61(9):588–603.

11. World Health Organization. Update of the Mental Health Gap Action Programme (mhGAP) guidelines for mental, neurological and substance use disorders [Internet]. 2015. Available from: https://www.who.int/maternal_child_adolescent/documents/health-promotion-interventions/en/

12. Haute Autorité de Santé. Recommandation de bonne pratique - Manifestations dépressives à l’adolescence: repérage, diagnostic et prise en charge en soins de premier recours [Internet]. Paris, France; 2014. Available from: https://www.has-sante.fr/portail/upload/docs/application/pdf/2014-12/manifestations_depressives_recommandations.pdf

13. Dolle K, Schulte-Körne G. The treatment of depressive disorders in children and adolescents. Dtsch Arztebl Int. 2013;110(50).

14. Dolle K, Schulte-Körne G. Komplementäre ansätze zur behandlung von depressiven störungen bei kindern und jugendlichen. Prax Kinderpsychol Kinderpsychiatr. 2014;63(3):237–63.

15. Ministerio de Salud. Guía Clínica Depresión en personas de 15 años y más [Internet]. Santiago, Chile; 2013 [cited 2020 Nov 1]. Available from: https://www.minsal.cl/portal/url/item/7222754637c08646e04001011f014e64.pdf

16. Ministerio de Salud. Guía Clínica para el tratamiento de adolescentes de 10 a 14 años con Depresión. [Internet]. Santiago, Chile; 2013 [cited 2020 Nov 1]. Available from: https://www.minsal.cl/portal/url/item/e11791fc480273e9e040010164014e60.pdf

17. Ministry of Health Singapore. Depression - MOH Clinical Practice Guidelines 1/2012 [Internet]. Singapore; 2012. Available from: https://www.moh.gov.sg/content/dam/moh_web/HPP/Doctors/cpg_medical/current/2012/depression/Depression CPG_R14_FINAL.pdf

18. Beyondblue. Clinical practice guidelines: depression in adolescents and young adults. 2011. 164 p.

19. Cincinnati Children’s Hospital Medical Centre. Best Evidence Statement (BESt): Treatment of children and adolescents with Major Depressive Disorder (MDD) during the Acute Phase. Cincinnati, OH; 2010.

20. Guidelines and Protocols Advisory Committee. Anxiety and Depression in Children and Youth - Diagnosis and Treatment [Internet]. British Columbia Guidelines & Protocols. 2010. Available from: https://www2.gov.bc.ca/assets/gov/health/practitioner-pro/bc-guidelines/depressyouth.pdf

21. US Preventive Services Task Force. Screening and treatment for major depressive disorder in children and adolescents: US Preventive Services Task Force recommendation statement. Pediatrics. 2009;123(4):1223–8.

22. Birmaher B, Brent D. Practice parameter for the assessment and treatment of children and adolescents with depressive disorders. J Am Acad Child Adolesc Psychiatry. 2007;46(11):1503–26.

23. Gallagher R. Evidence ­ Based Psychotherapies for Depressed Adolescents: A Review and Clinical Guidelines. Prim Psychiatry. 2005;12(9):33.

24. Bostic JQ, Rubin DH, Prince J, Schlozman S. Treatment of Depression in Children and Adolescents. J Psychiatr Pract. 2005;11(3):141–54.
