# Supplementary material for: Problem-solving training as an active ingredient of treatment for youth depression: a scoping review and exploratory meta-analysis
Source: BMC Psychiatry. 2021 Aug 24;21:397. doi: 10.1186/s12888-021-03260-9 (PMC8383463; doi:10.1186/s12888-021-03260-9)
Supplement: Supplementary file 5 — Additional file 5. Additional Data and Outputs from the Meta-Analysis. [file 12888_2021_3260_MOESM5_ESM.docx]

#

Problem-Solving Training as an Active Ingredient of Treatment for Youth Depression: A Scoping Review and Exploratory Meta-Analysis

**ADDITIONAL FILE 5**

**Additional Data and Outputs from the Meta-Analysis**

Karolin R. Krause^1,2^, Darren B. Courtney^1,3^, Benjamin W. C. Chan^4^, Sarah Bonato^1^, Madison Aitken^1,3^, Jacqueline Relihan^1^, Matthew Prebeg^1^, Karleigh Darnay^1^, Lisa D. Hawke^1,3^, Priya Watson^1,3^, Peter Szatmari^1,3,5^

1. Cundill Centre for Child and Youth Depression, Centre for Addiction and Mental Health (CAMH), Toronto, ON, Canada
2. Evidence-Based Practice Unit, University College London and Anna Freud National Centre for Children and Families and, London, United Kingdom
3. Department of Psychiatry, University of Toronto, Toronto, ON, Canada
4. Department of Family and Community Medicine, University of Toronto, Toronto, ON, Canada
5. Hospital for Sick Children, Toronto, ON, Canada

**Corresponding Author:** Karolin Krause, Cundill Centre for Child and Youth Depression, Centre for Addiction and Mental Health, 80 Workman Way, Toronto, ON M6J 1H4, Canada; Email: Karolin.krause@camh.ca

# Additional Outputs from the Meta-Analysis

Fig S1. Forest Plot: Random Effects Model with Self-Reported Depression or Emotional Symptom Severity as the Outcome (Continuous) – Excluding Studies with High Risk of Bias

| 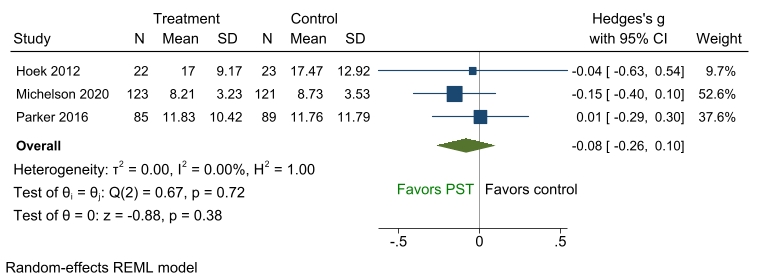 |
| --- |

Fig S2. Forest Plot: Random Effects Model with Clinician-rated Depression Severity as the Outcome (Continuous; k =2)

| 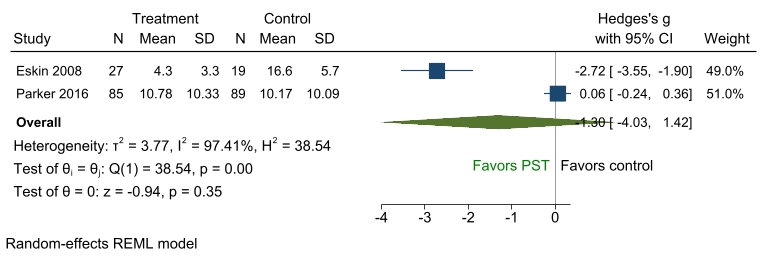 |
| --- |

Fig S3. Funnel Plot of Standard Error by Hedges' g

| 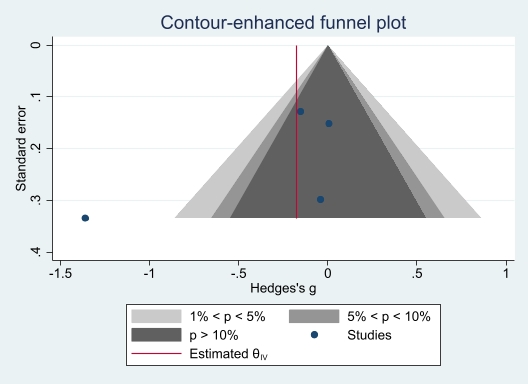 |
| --- |
