# Supplementary material for: Problem-solving training as an active ingredient of treatment for youth depression: a scoping review and exploratory meta-analysis
Source: BMC Psychiatry. 2021 Aug 24;21:397. doi: 10.1186/s12888-021-03260-9 (PMC8383463; doi:10.1186/s12888-021-03260-9)
Supplement: Supplementary file 6 — Additional file 6. Risk of Bias Assessment and GRADE Appraisal. [file 12888_2021_3260_MOESM6_ESM.docx]

Problem-Solving Training as an Active Ingredient of Treatment for Youth Depression: A Scoping Review and Exploratory Meta-Analysis

**ADDITIONAL FILE 6**

**Risk of Bias Assessment and GRADE Appraisal**

Karolin R. Krause^1,2^, Darren B. Courtney^1,3^, Benjamin W. C. Chan^4^, Sarah Bonato^1^, Madison Aitken^1,3^, Jacqueline Relihan^1^, Matthew Prebeg^1^, Karleigh Darnay^1^, Lisa D. Hawke^1,3^, Priya Watson^1,3^, Peter Szatmari^1,3,5^

1. Cundill Centre for Child and Youth Depression, Centre for Addiction and Mental Health (CAMH), Toronto, ON, Canada
2. Evidence-Based Practice Unit, University College London and Anna Freud National Centre for Children and Families and, London, United Kingdom
3. Department of Psychiatry, University of Toronto, Toronto, ON, Canada
4. Department of Family and Community Medicine, University of Toronto, Toronto, ON, Canada
5. Hospital for Sick Children, Toronto, ON, Canada

**Corresponding Author:** Karolin Krause, Cundill Centre for Child and Youth Depression, Centre for Addiction and Mental Health, 80 Workman Way, Toronto, ON M6J 1H4, Canada; Email: Karolin.krause@camh.ca

**Risk of Bias Assessment**

**Table S2. Results from ROB Assessment Using the Cochrane ROB2 Tool** (1) **(Studies with Intention-to-Treat)**

| **Study** | **Outcome** | **Randomisation Process** | **Deviations from intended interventions** | **Missing Outcome Data** | **Measurement of the outcome** | **Selection of reported results** | **Overall bias** |
| --- | --- | --- | --- | --- | --- | --- | --- |
| Eskin et al. (2008) | BDI: Self-reported depressive symptoms (continuous) | Some concerns | Low | High | Some concerns | High | High |
| Eskin et al. (2008) | BDI: Recovery from depressive symptoms (dichotomous) | Some concerns | Low | High | Some concerns | High | High |
| Eskin et al. (2008) | HDRS: Clinician-rated depressive symptoms (continuous) | Some concerns | Low | High | Some concerns | High | High |
| Eskin et al. (2008) | HDRS: Clinician-rated depressive symptoms - recovery (dichotomous) | Some concerns | Low | High | Some concerns | High | High |
| Hoek et al. (2012) | Depressive symptoms (CES-D) – summary score (continuous) | Low | Low | Some concerns | Some concerns | Low | Some concerns |
| Hoek et al. (2012) | Depressive symptoms (CES-D) – Recovery (dichotomous) | Low | Low | Some concerns | Some concerns | Low | Some concerns |
| Hoek et al. (2012) | Hospital Anxiety and Depression Scale, Anxiety Subscale – summary score (continuous) | Low | Low | Some concerns | Low | Low | Some concerns |
| Hoek et al. (2012) | Hospital Anxiety and Depression Scale, Anxiety Subscale – recovery (dichotomous) | Low | Low | Some concerns | Low | Low | Some concerns |
| Parker et al. (2016) | BDI-II: Self-reported depressive symptoms post intervention (continuous) | Low | Low | Some concerns | Low | Low | Some concerns |
| Parker et al. (2016) | MADRS: Observer-rated depressive symptoms post treatment (continuous) | Low | Low | Some concerns | Low | Low | Some concerns |
| Parker et al. (2016) | BAI: Self-reported anxiety symptoms post treatment (continuous) | Low | Low | Some concerns | Low | Low | Some concerns |
| Michelson et al. (2020) | SDQ Total Difficulties score: severity of self-reported mental health symptoms at 6 weeks (continuous) | Low | Low | Low | Low | Low | Low |
| Michelson et al. (2020) | Youth Top Problems (YTP) score: idiographic top problems at 6 weeks (continuous) | Low | Low | Low | Low | Low | Low |
| Michelson et al. (2020) | SDQ internalising score: internalising symptoms at 6 weeks (continuous) | Low | Low | Low | Low | Low | Low |

**Table S3. ROB Rating Rationales**

| **Study** | **Overall ROB Rating** | **ROB rating rationale** |
| --- | --- | --- |
| Eskin et al. (2008) (2) | High risk of bias | Lack of preregistration of the trial (the article provides no reference to a trial protocol or analysis plan, and an attempt to obtain these from the study authors was unsuccessful), comparison with a waitlist condition with high dropout, potential for participant expectancy/placebo effects to have influenced self-reported outcome ratings compared with a non-active control group, and lack of adequate missing data handling are issues that most likely to contribute to bias. |
| Hoek et al. (2012) (3) | Some concerns | The main concerns are due to missingness of outcome data and not knowing if this is related to symptom severity, and participants being aware of their assignment to active treatment vs waitlist control, which could create expectancy effects on self-reported outcome ratings. The presence of a trial protocol and best available methods for handling missing data are strengths. |
| Parker et al. (2016) (4) | Some concerns | The existence of a trial protocol, use of a standard measure of depression, intention-to-treat analysis, and active treatment comparison conditions are strengths that reduce bias, including bias that may arise from using self-report for outcome measurement in non-blinded designs (due to expectancy effects). Missing outcome data (although handled appropriately) is a weakness. |
| Michelson et al. (2020) (5) | Low risk of bias | Overall this is a thoroughly planned trial with a protocol and rigorous adherence to masking. The risk of expectancy effects due to self-report on outcomes in the absence of blinding (which is generally not possible in psychosocial interventions) is mitigated by the control condition receiving problem-solving support via self-help booklets (rather than being waitlisted). |

**GRADE Assessment**

**Table S4. GRADE Summary of Findings Table (6)**

| **Problem-Solving Therapy Compared to Waitlist or Control Conditions for Youth Depression** | | | | | | |
| --- | --- | --- | --- | --- | --- | --- |
| **Patient or population**: Youth Depression (ages 14-24 years)  **Setting**: Clinical or community settings  **Intervention**: Problem-Solving Therapy (PST)  **Comparison**: Waitlist or control condition | | | | | | |
| Outcomes | **Anticipated absolute effects^*^** (95% CI) | | Relative effect (95% CI) | № of participants  (studies) | Certainty of the evidence (GRADE) | Comments |
|  | **Risk with Control Condition** | **Risk with Problem solving therapy** |  |  |  |  |
| **Primary Outcome of this Meta-Analysis** | | | | | | |
| Depression severity assessed via self-report with: BDI-I; BDI-II; CES-D; SDQ Emotions follow up: median 6 weeks | — | Hedges' g **0.34 lower** (0.92 lower to 0.23 higher) | — | 509 (4 RCTs) | ⨁◯◯◯ VERY LOW ^a^ | Problem solving therapy may reduce depression severity, but the evidence is and of very low quality. |
| Depression severity – restricted to studies with less than high risk of bias follow up: median 6 weeks | — | Hedges' g **0.08 lower** (0.26 lower to 0.10 higher) | — | 509 (4 RCTs) | ⨁⨁◯◯ VERY LOW ^b^ | Problem solving therapy may have little to no effect on depression severity, but the evidence is uncertain. |
| **Secondary Outcomes** | | | | | | |
| Depression severity (clinician-reported) assessed with: HADS; MADRS follow up: 6 weeks | — | Hedges' g **1.30 lower** (4.03 lower to 1.42 higher) | —- | 220  (2 RCTs) | ⨁◯◯◯ VERY LOW ^c^ | Data pooled from two studies. |
| Personal Problems assessed with: Youth Top Problems Scale follow up: 6 weeks | — | Adjusted effect size **0.36 lower** (0.11 lower to 0.61 lower) | — | 250 (1 RCT) | ⨁⨁◯◯ LOW ^d^ | Data as reported in a single study. |
| Functional impairment assessed with: SDQ Impact Supplement follow up: mean 12 weeks | — | Adjusted mean difference **0.86 lower** (2.62 lower to 0.9 higher) | — | 243 (1 RCT) | ⨁⨁◯◯ LOW ^d^ | Data as reported in a single study. |
| ***The risk in the intervention group** (and its 95% confidence interval) is based on the assumed risk in the comparison group and the **relative effect** of the intervention (and its 95% CI).  **CI:** Confidence interval | | | | | | |
| **GRADE Working Group grades of evidence** **High certainty:** We are very confident that the true effect lies close to that of the estimate of the effect **Moderate certainty:** We are moderately confident in the effect estimate: The true effect is likely to be close to the estimate of the effect, but there is a possibility that it is substantially different **Low certainty:** Our confidence in the effect estimate is limited: The true effect may be substantially different from the estimate of the effect **Very low certainty:** We have very little confidence in the effect estimate: The true effect is likely to be substantially different from the estimate of effect | | | | | | |

**Explanations**

a. Downgraded by 4 for risk of bias, inconsistency, indirectness, imprecision. Risk of bias: We rated one study has having high risk of bias, two studies as having some risk of bias, and only one study as having a low risk of bias. Inconsistency: Heterogeneity is > 75%. A large part of the heterogeneity is reduced when excluding the one study with high risk of bias (Eskin et al., 2008) from the meta-analysis. Indirectness: There are concerns of indirectness related to considerable differences in the study populations, with only one study requiring a clinical diagnosis of depression, one study requiring above-threshold depression or anxiety symptoms, and two studies requiring above-threshold mental health difficulties. Although at least 50% of participants in all studies showed above-threshold depression symptoms or emotional difficulties, the study samples were not directly representative of youth with clinical depression. Imprecision: The pooled sample size is below the minimum sample size required as per a power calculation for a T-test of independent means (two-tailed) with alpha and beta errors set at 0.05 and 0.2, and an expected effect size of 0.2. For three out of four studies and the pooled effect, the confidence interval includes no effect.

b. Downgraded by 2 for indirectness and imprecision.

c. Downgraded by 4 for risk of bias, severe inconsistency, and severe imprecision.

d. Downgraded by 2 for indirectness and imprecision.

**References**

1. Higgins JP, Savović J, Page MJ, Sterne JA, Group on behalf of the RD. Guidance: RoB 2: A revised tool for assessing risk of bias in randomised trials. BMJ. 2019;366(August).

2. Eskin M, Ertekin K, Demir H. Efficacy of a Problem-Solving Therapy for Depression and Suicide Potential in Adolescents and Young Adults. Cognit Ther Res. 2008;32(2):227–45.

3. Hoek W, Schuurmans J, Koot HM, Cuijpers P. Effects of Internet-Based Guided Self-Help Problem-Solving Therapy for Adolescents with Depression and Anxiety: A Randomized Controlled Trial. PLoS One. 2012;7(8):1–7.

4. Parker AG, Hetrick SE, Jorm AF, Mackinnon AJ, McGorry PD, Yung AR, et al. The effectiveness of simple psychological and physical activity interventions for high prevalence mental health problems in young people: A factorial randomised controlled trial. J Affect Disord. 2016 May;196:200–9.

5. Michelson D, Malik K, Parikh R, Weiss HA, Doyle AM, Bhat B, et al. Effectiveness of a brief lay counsellor-delivered, problem-solving intervention for adolescent mental health problems in urban, low-income schools in India: a randomised controlled trial. Lancet Child Adolesc Heal. 2020 Aug;4(8):571–82.

6. GRADEpro GDT. GRADEpro Guideline Development Tool [Software] [Internet]. McMaster University (developed by Evidence Prime, Inc.); 2020. Available from: gradepro.org
