# Supplementary material for: Problem-solving training as an active ingredient of treatment for youth depression: a scoping review and exploratory meta-analysis
Source: BMC Psychiatry. 2021 Aug 24;21:397. doi: 10.1186/s12888-021-03260-9 (PMC8383463; doi:10.1186/s12888-021-03260-9)
Supplement: Supplementary file 7 — Additional file 7. Illustration of Insights from the Consultation of Youth Advisors. [file 12888_2021_3260_MOESM7_ESM.docx]

Problem-Solving Training as an Active Ingredient of Treatment for Youth Depression: A Scoping Review and Exploratory Meta-Analysis

**ADDITIONAL FILE 7**

**Illustration of Insights from the Consultation of Youth Advisors**

Karolin R. Krause^1,2^, Darren B. Courtney^1,3^, Benjamin W. C. Chan^4^, Sarah Bonato^1^, Madison Aitken^1,3^, Jacqueline Relihan^1^, Matthew Prebeg^1^, Karleigh Darnay^1^, Lisa D. Hawke^1,3^, Priya Watson^1,3^, Peter Szatmari^1,3,5^

1. Cundill Centre for Child and Youth Depression, Centre for Addiction and Mental Health (CAMH), Toronto, ON, Canada
2. Evidence-Based Practice Unit, University College London and Anna Freud National Centre for Children and Families and, London, United Kingdom
3. Department of Psychiatry, University of Toronto, Toronto, ON, Canada
4. Department of Family and Community Medicine, University of Toronto, Toronto, ON, Canada
5. Hospital for Sick Children, Toronto, ON, Canada

**Corresponding Author:** Karolin Krause, Cundill Centre for Child and Youth Depression, Centre for Addiction and Mental Health, 80 Workman Way, Toronto, ON M6J 1H4, Canada; Email: Karolin.krause@camh.ca

Fig S4. Proposed Principles for Youth-Friendly Problem-Solving Training Based on the Review Team’s Consultation with Youth Advisors

| 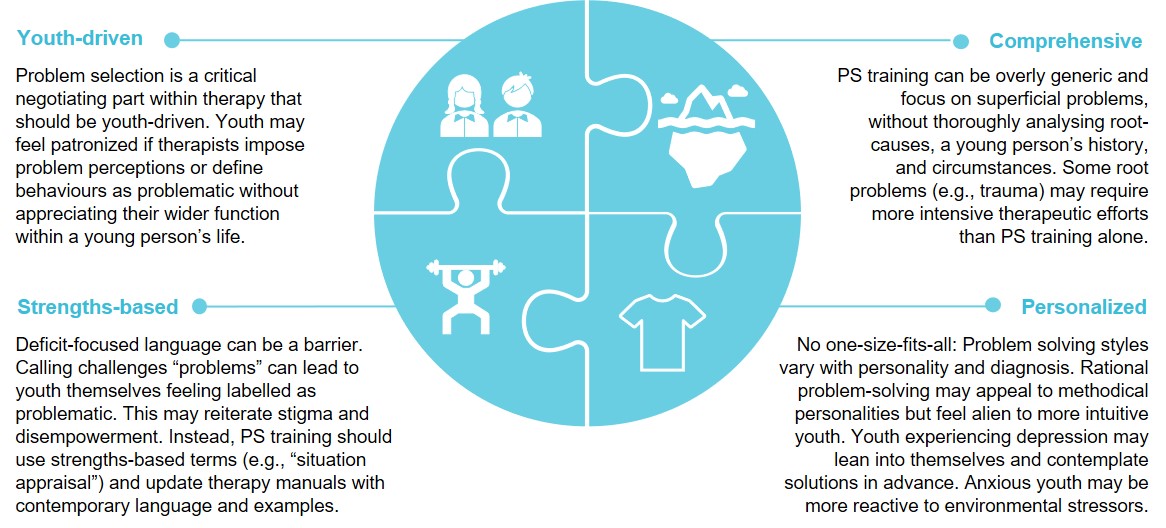 |
| --- |
